# Supplementary material for: Synthesis, Antimalarial, Antileishmanial, and Cytotoxicity Activities and Preliminary In Silico ADMET Studies of 2-(7-Chloroquinolin-4-ylamino)ethyl Benzoate Derivatives
Source: Pharmaceuticals (Basel). 2023 Dec 9;16(12):1709. doi: 10.3390/ph16121709 (PMC10747975; doi:10.3390/ph16121709)
Supplement: Supplementary file 1 [file pharmaceuticals-16-01709-s001.zip › pharmaceuticals-2668748-supplementary.pdf]

Supplementary data

Synthesis, antimalarial, antileishmanial, and cytotoxicity activities, and preliminary *in silico* ADMET studies of 2-(7-chloroquinolin-4-ylamino)ethyl benzoate derivatives

Joyce E. Gutiérrez [1], Hegira Ramírez [2,*], Esteban Fernandez-Moreira [3], María E. Acosta [4], Michael R. Mijares [5], Juan Bautista De Sanctis [6], Soňa Gurská [6], Petr Džubák [6], Marián Hajdúch [6], Liesangerli Labrador-Fagúndez [7], Bruno G. Stella [7], Luis José Díaz-Pérez [7], Gustavo Benaim [7,8] and Jaime E. Charris [1,*]

[1]Organic Synthesis Laboratory, Faculty of Pharmacy, Central University of Venezuela, Los Chaguaramos 1041-A, Caracas 47206, Venezuela; gutierrez.joyce@gmail.com

[2] Facultad de Ciencias de la Salud y Desarrollo Humano, Universidad Ecotec, Km. 13.5 Samborondón, Samborondón, Guayas, Guayaquil, EC092302, Ecuador

[3]Escuela de Medicina, Universidad Espíritu Santo, Samborondón, Guayas, Guayaquil, E092301, Ecuador; estebanfernandez@uees.edu.ec

[4]Unidad de Bioquímica, Facultad de Farmacia, Universidad Central de Venezuela, Los Chaguaramos 1041-A, Caracas 47206, Venezuela; mariuacosta0103@gmail.com

[5]Biotechnology Unit, Faculty of Pharmacy, Central University of Venezuela, 47206, Los Chaguaramos 1041-A, Caracas, Venezuela; michaelfarmacia@gmail.com

[6]Institute of Molecular and Translational Medicine, Faculty of Medicine and Dentistry, Palacky University, Hněvotínská 1333/5, Olomouc 779 00, Czech Republic; juanbautista.desanctis@upol.cz (JDS); sona.gurska@upol.cz (SG); petr.dzubak@upol.cz (PD); marian.hajduch@upol.cz (MH)

[7]Unidad de Bioquímica de Parásitos y Señalización Celular. Instituto de Estudios Avanzados (IDEA). Caracas. Venezuela

[8]Instituto de Biología Experimental. Facultad de Ciencias. Universidad Central de Venezuela. Caracas. Venezuela. gbenaim@gmail.com

Table of Contents

		Page
**S1**	The IR spectrum of 2	4
**S2**	The ^{1}H NMR spectrum of 2	4
**S3**	The ^{13}C NMR spectrum of 2	5
**S4**	The DEPT 135° spectrum of 2	5
**S5**	The IR spectrum of 4a	6
**S6**	The ^{1}H NMR spectrum of 4a	6
**S7**	The ^{13}C NMR spectrum of 4a	7
**S8**	The DEPT 135° spectrum of 4a	7
**S9**	The COSY spectrum of 4a	8
**S10**	The HETCOR spectrum of 4a	8
**S11**	The IR spectrum of 4b	9
**S12**	The ^{1}H NMR spectrum of 4b	9
**S13**	The ^{13}C NMR spectrum of 4b	10
**S14**	The DEPT 135° spectrum of 4b	10
**S15**	The IR spectrum of 4c	11
**S16**	The ^{1}H NMR spectrum of 4c	11
**S17**	The ^{13}C NMR spectrum of 4c	12
**S18**	The DEPT 135° spectrum of 4c	12
**S19**	The COSY spectrum of 4c	13
**S20**	The HETCOR spectrum of 4c	13
**S21**	The IR spectrum of 4d	14
**S22**	The ^{1}H NMR spectrum of 4d	14
**S23**	The ^{13}C NMR spectrum of 4d	15
**S24**	The IR spectrum of 4e	16
**S25**	The ^{1}H NMR spectrum of 4e	16
**S26**	The ^{13}C NMR spectrum of 4e	16
**S27**	The DEPT 135° spectrum of 4e	17
**S28**	The IR spectrum of 4f	18
**S29**	The ^{1}H NMR spectrum of 4f	18
**S30**	The ^{13}C NMR spectrum of 4f	19
**S31**	The DEPT 135° spectrum of 4f	19
**S32**	The COSY spectrum of 4f	20
**S33**	The IR spectrum of 4g	21
**S34**	The ^{1}H NMR spectrum of 4g	21
**S35**	The ^{13}C NMR spectrum of 4g	22
**S36**	The DEPT 135° spectrum of 4g	22
**S37**	The COSY spectrum of 4g	23
**S38**	The HETCOR spectrum of 4g	23
**S39**	The IR spectrum of 4h	24
**S40**	The ^{1}H NMR spectrum of 4h	24
**S41**	The ^{13}C NMR spectrum of 4h	25

**S42**	The DEPT 135° spectrum of 4h	25
**S43**	The COSY spectrum of 4h	26
**S44**	The IR spectrum of 4i	27
**S45**	The ^{1}H NMR spectrum of 4i	27
**S46**	The ^{13}C NMR spectrum of 4i	28
**S47**	The DEPT 135° spectrum of 4i	28
**S48**	The COSY spectrum of 4i	29
**S49**	The IR spectrum of 4j	30
**S50**	The ^{1}H NMR spectrum of 4j	30
**S51**	The ^{13}C NMR spectrum of 4j	31
**S52**	The DEPT 135° spectrum of 4j	31
**S53**	The IR spectrum of 4l	32
**S54**	The ^{1}H NMR spectrum of 4l	32
**S55**	The ^{13}C NMR spectrum of 4l	33
**S56**	The DEPT 135° spectrum of 4l	33
**S57**	The COSY spectrum of 4l	34
**S58**	The HETCOR spectrum of 4l	34
**S59**	The IR spectrum of 4m	35
**S60**	The ^{1}H NMR spectrum of 4m	35
**S61**	The ^{13}C NMR spectrum of 4m	36
**S62**	The DEPT 135° spectrum of 4m	36
**S63**	19F NMR 4m	37

S1. Comp. 2

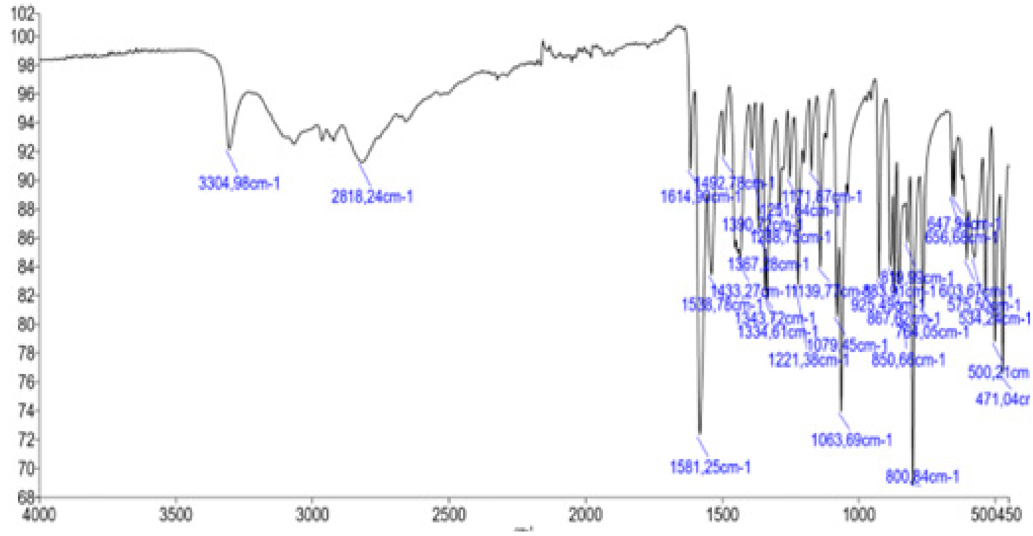

S2. Comp. 2

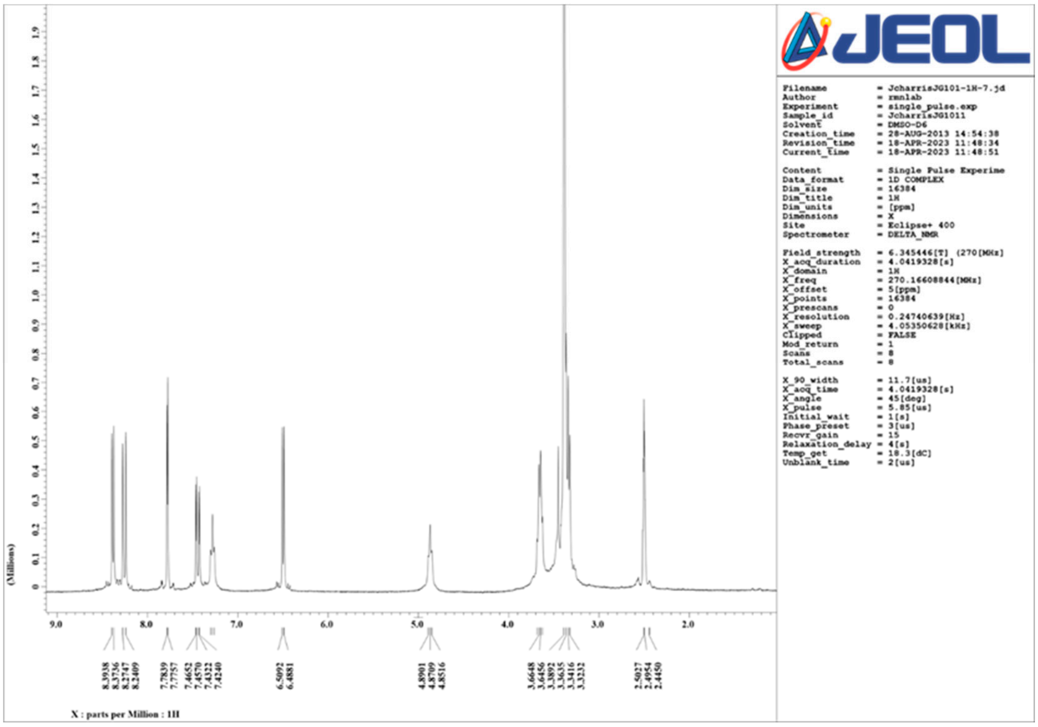

S3. Comp. 2

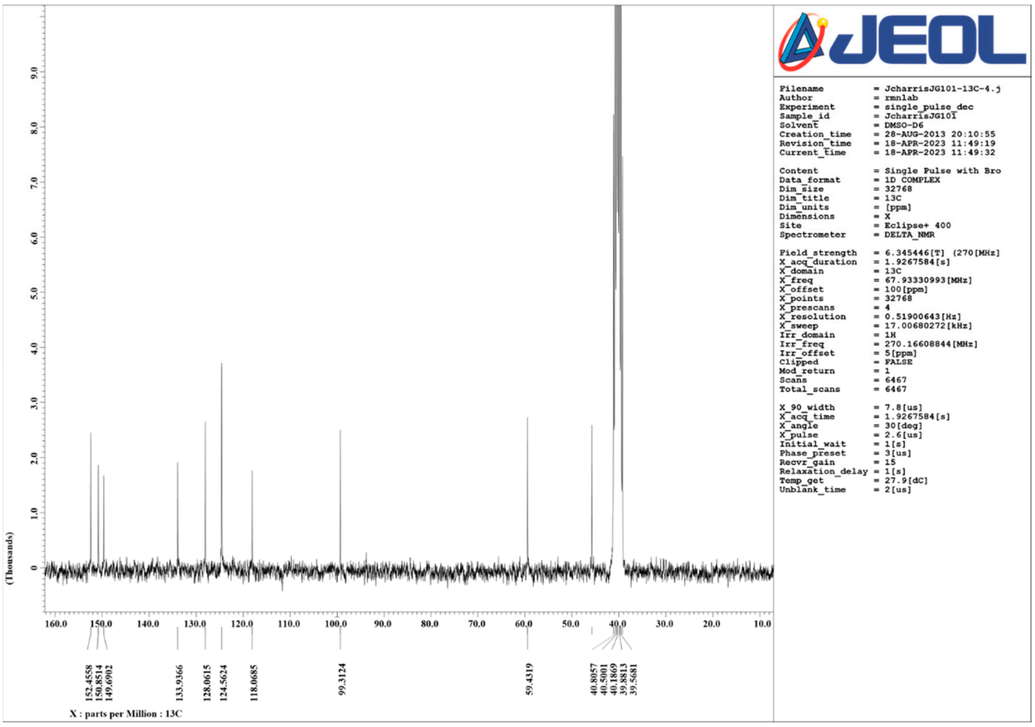

S4. Comp.2

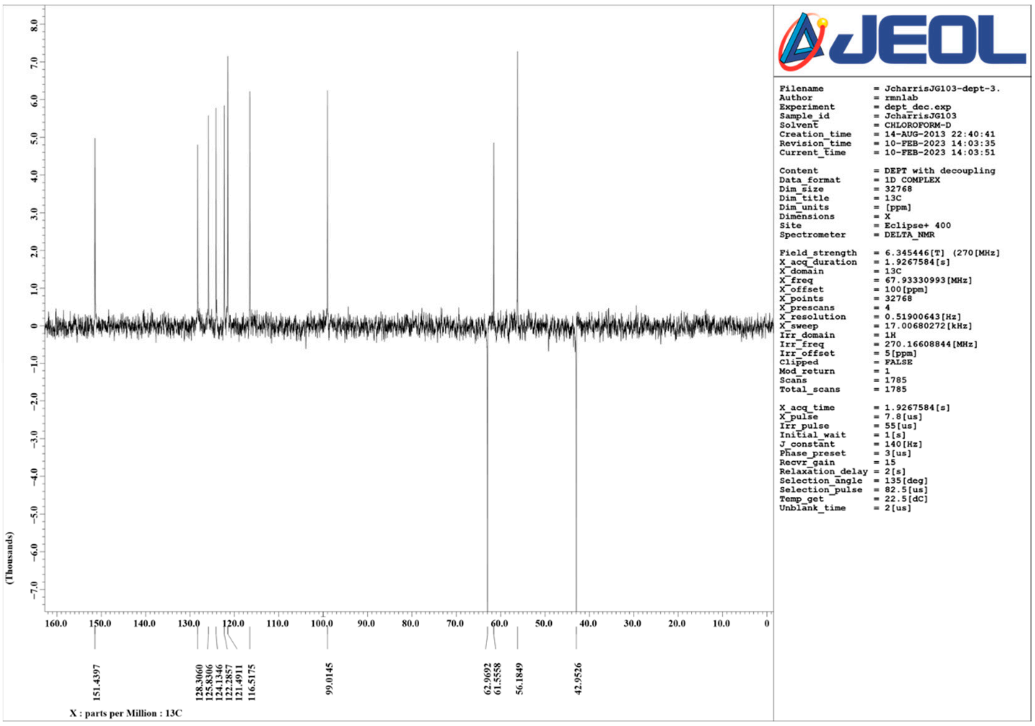

S5. Comp. 4a

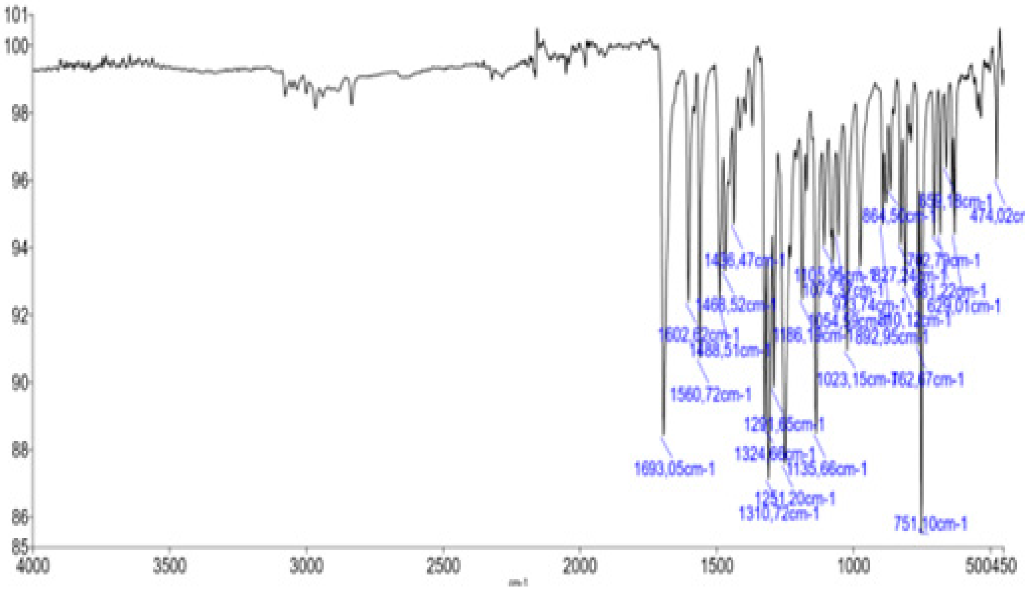

S6. Comp. 4a

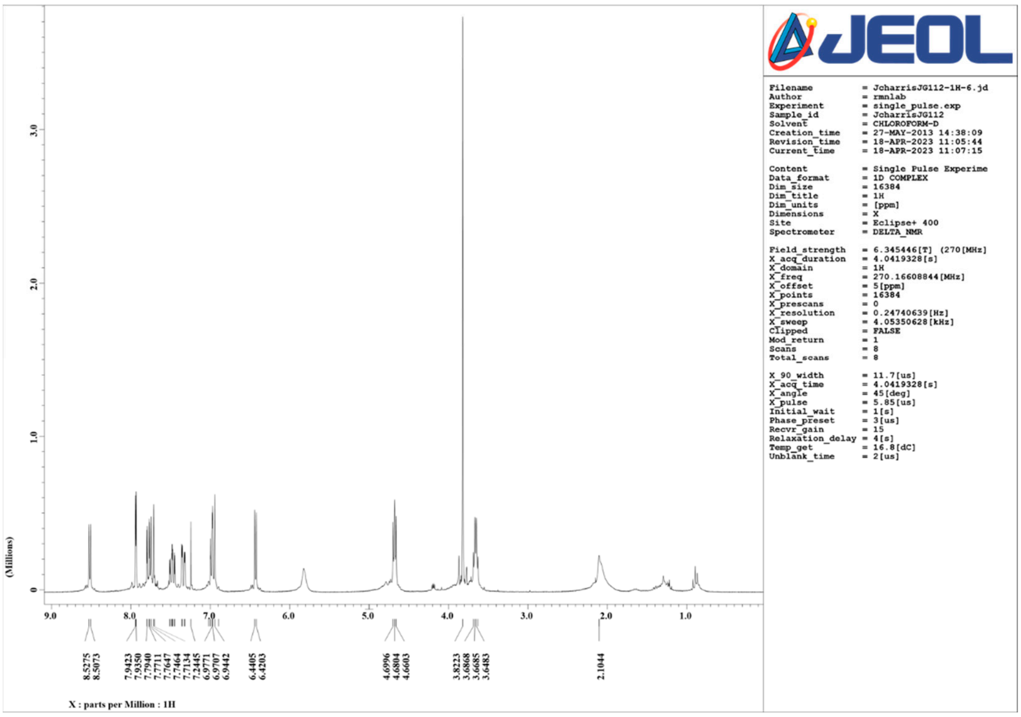

S7. Comp. 4a

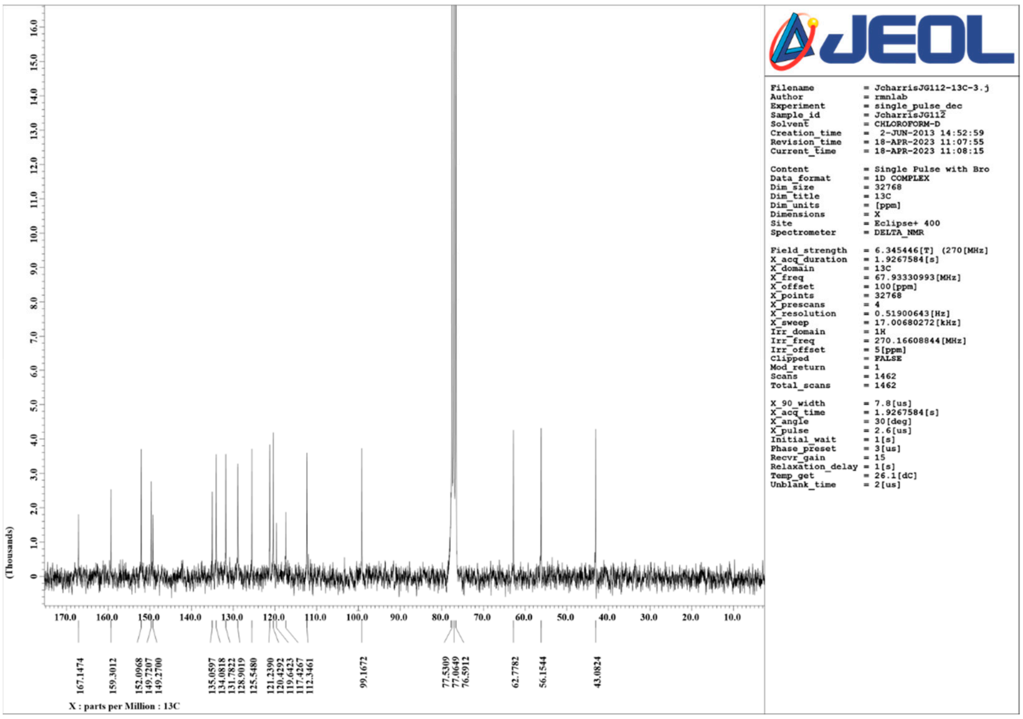

S8. Comp. 4a

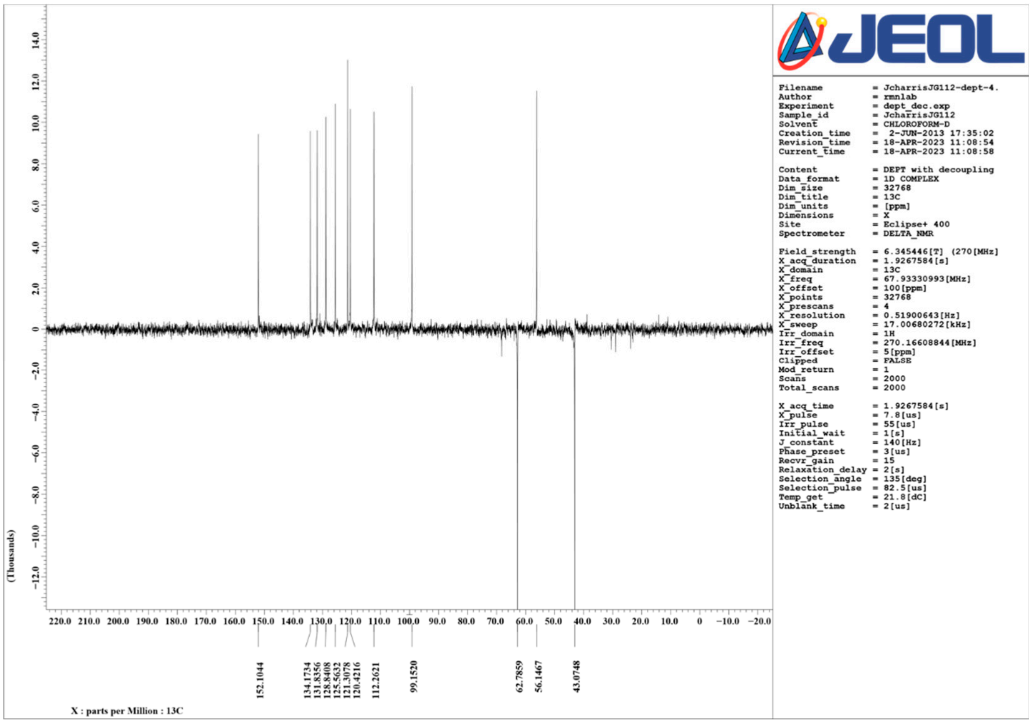

S9. Comp. 4a

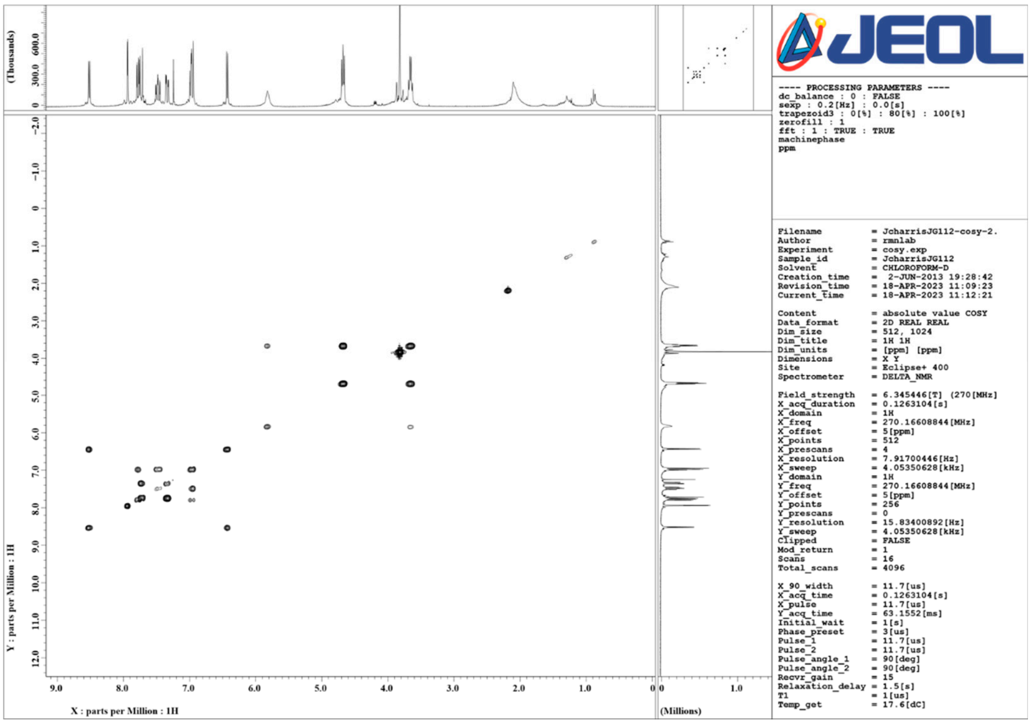

S10. Comp. 4a

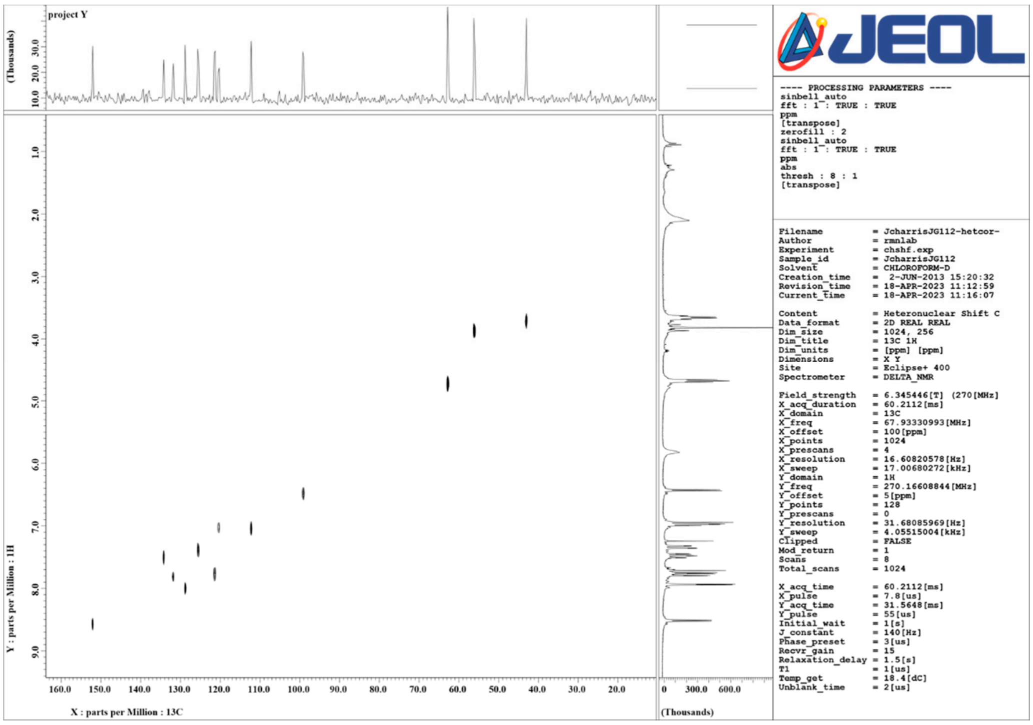

S11. Comp. 4b

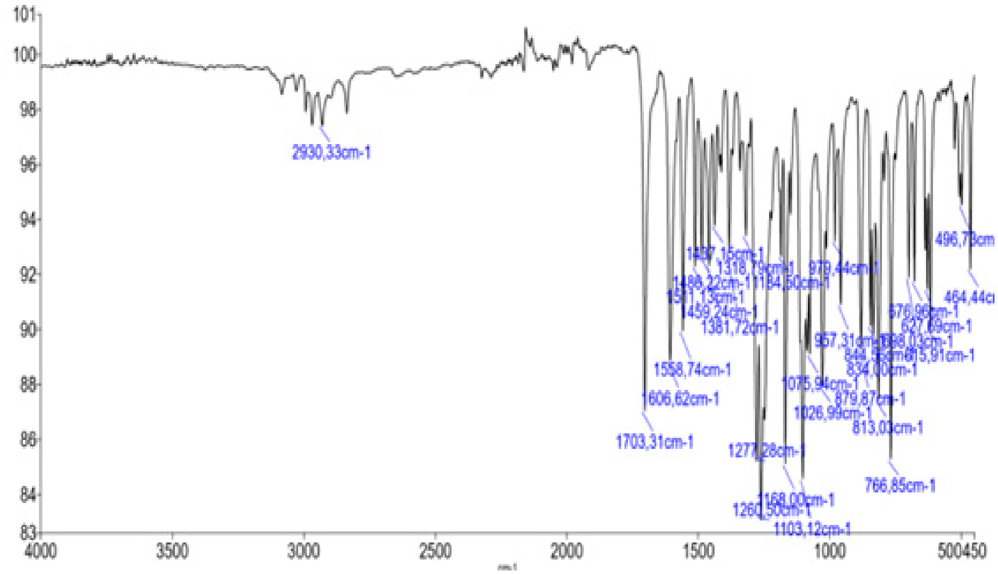

S12. Comp. 4b

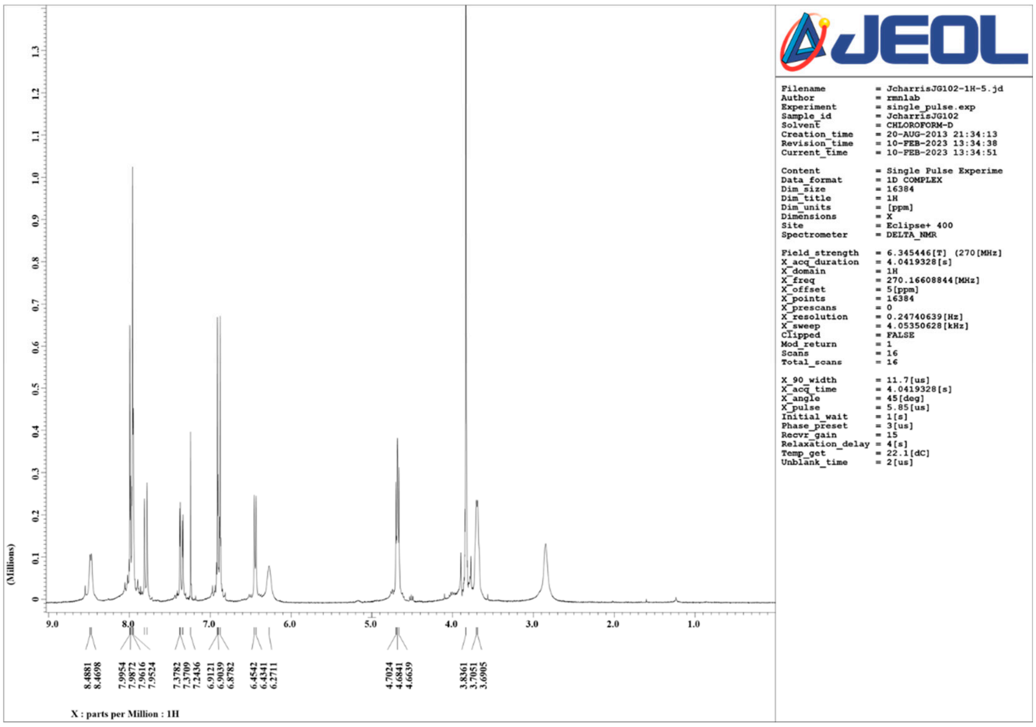

S13. Comp. 4b

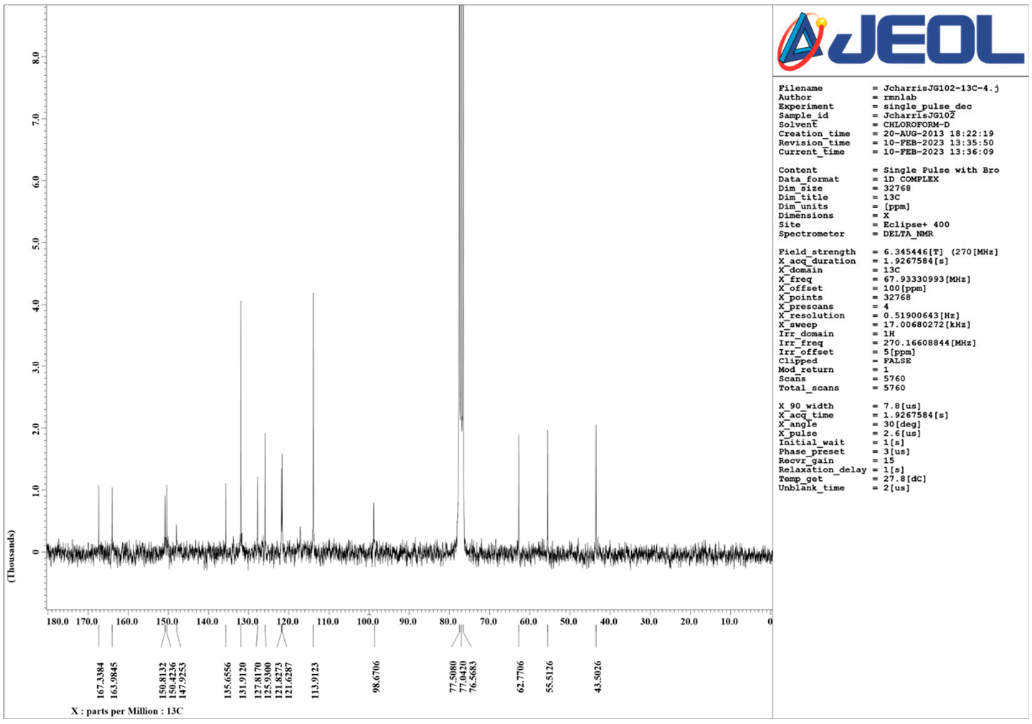

S14. Comp. 4b

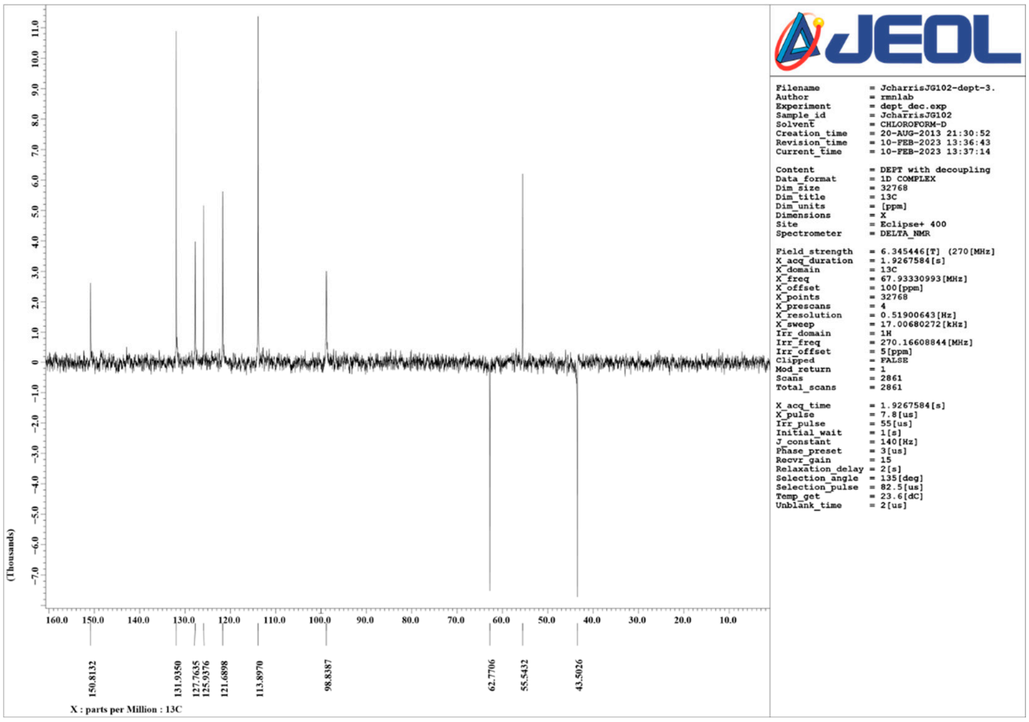

S15. Comp. 4c

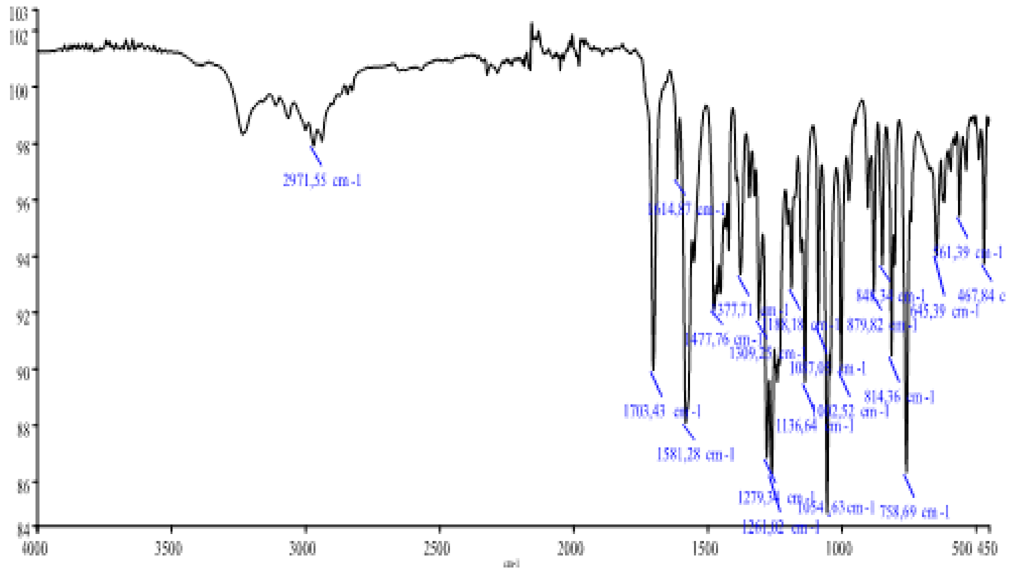

S16. Comp. 4c

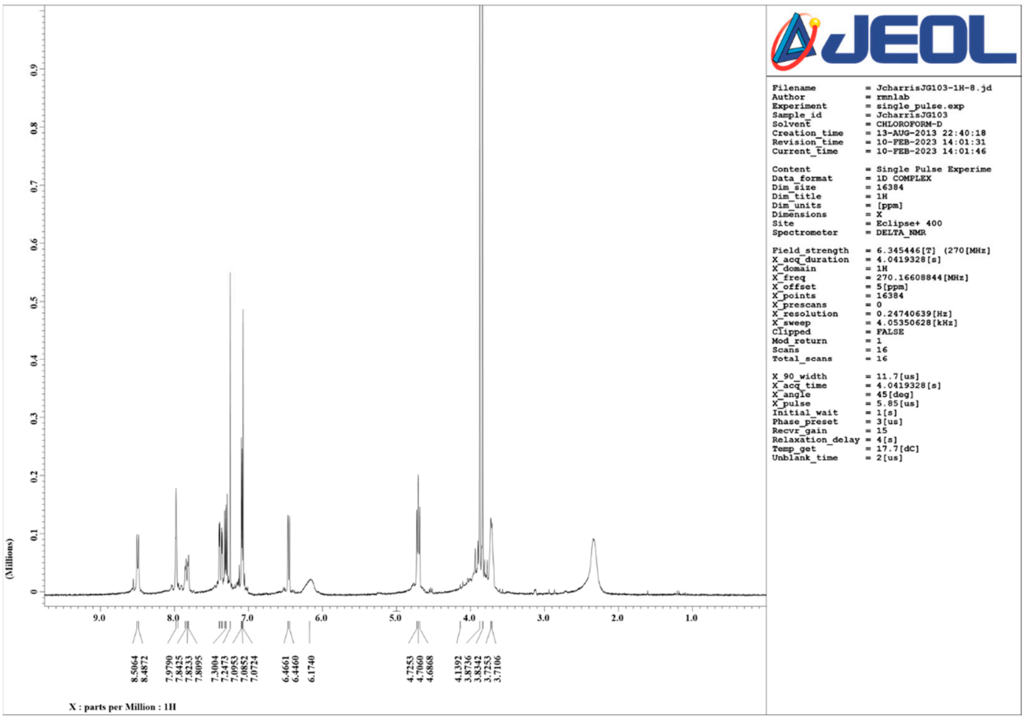

S17. Comp. 4c

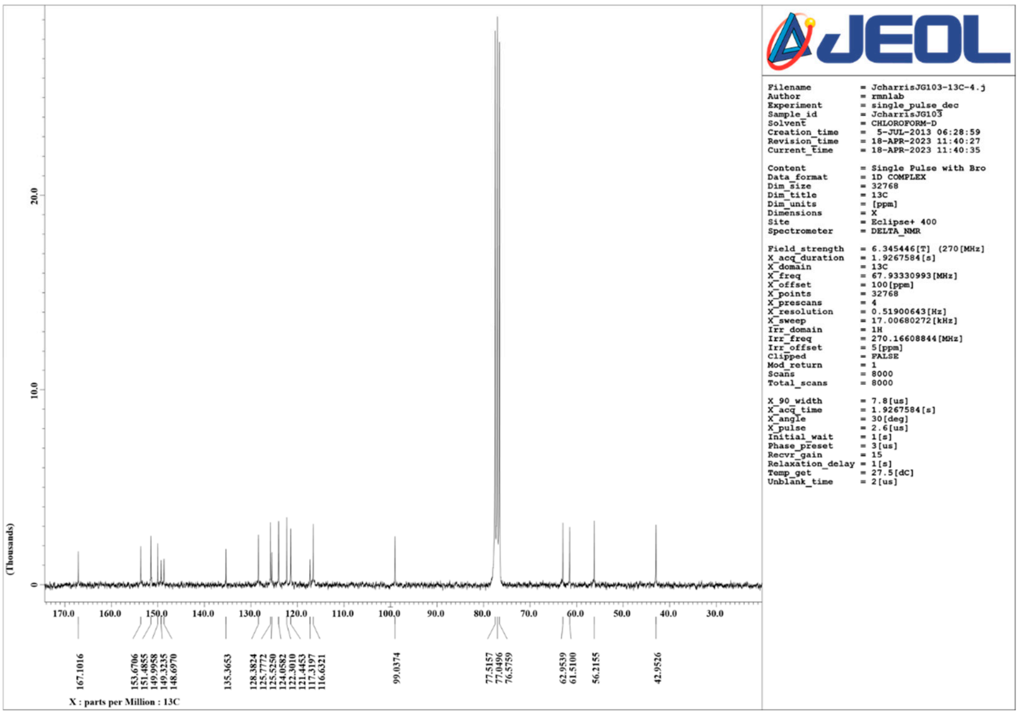

S18. Comp. 4c

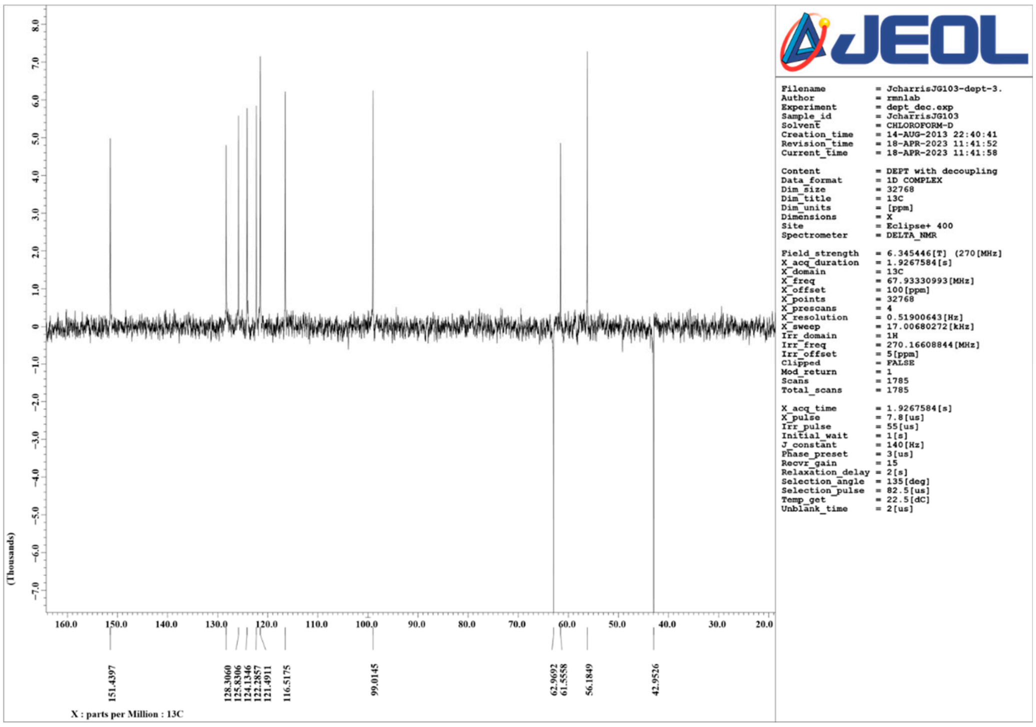

S19. Comp. 4c

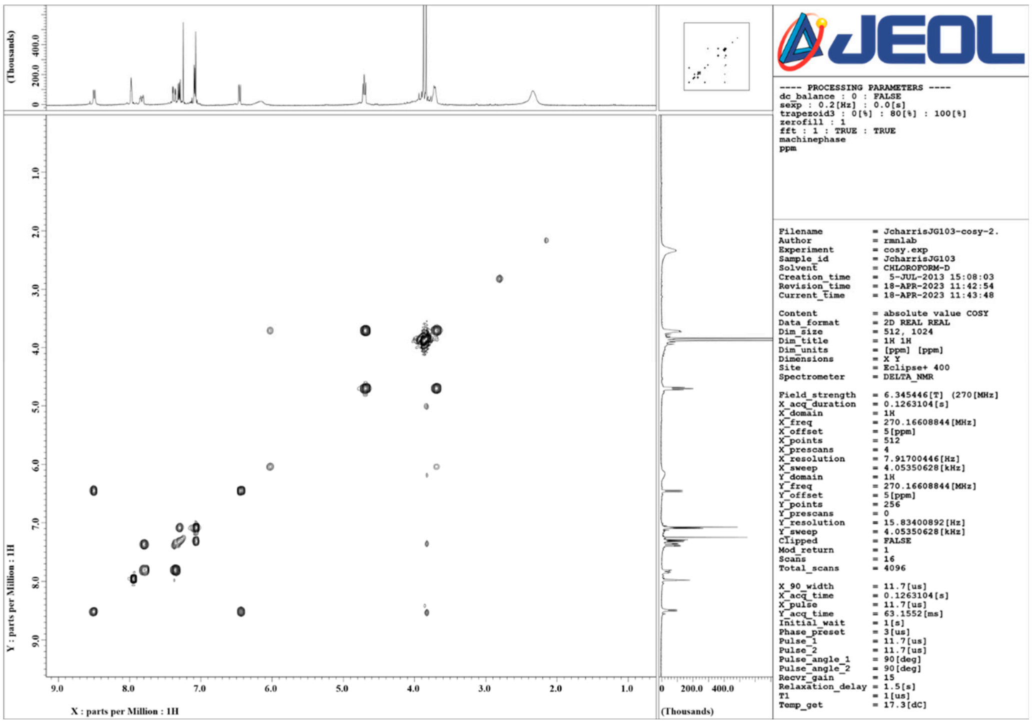

S20. Comp. 4c

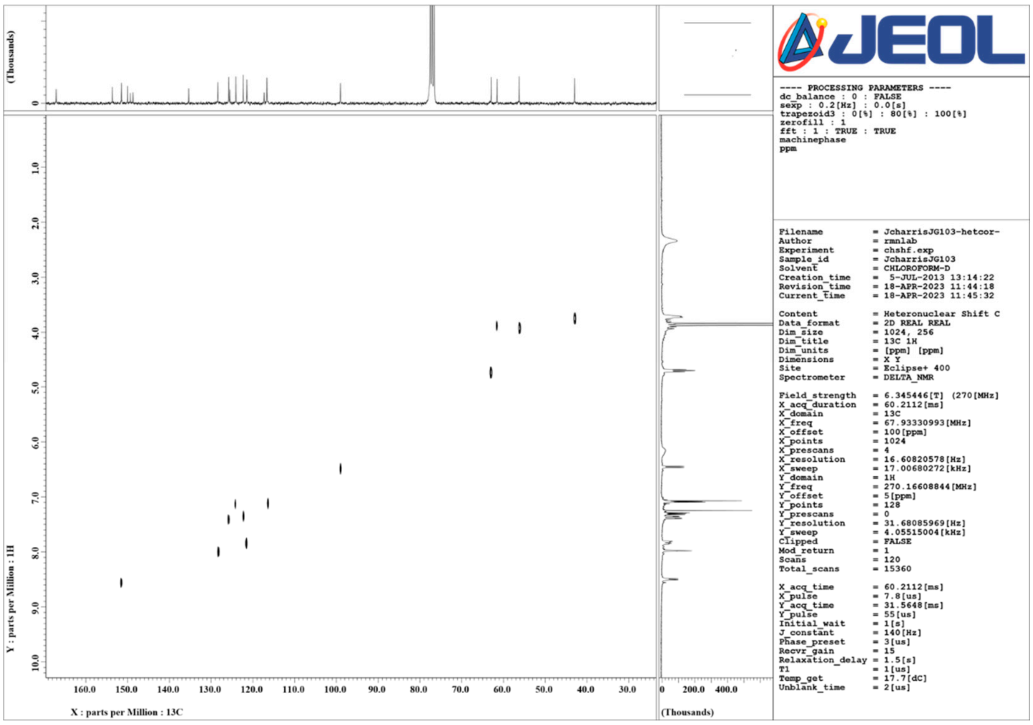

S21. Comp. 4d

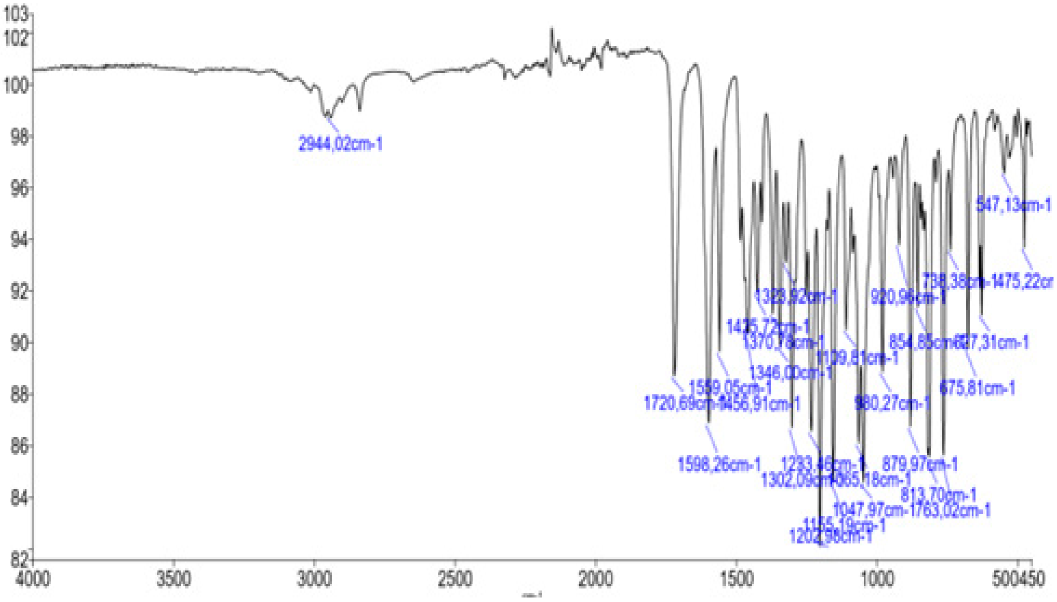

S22. Comp. 4d

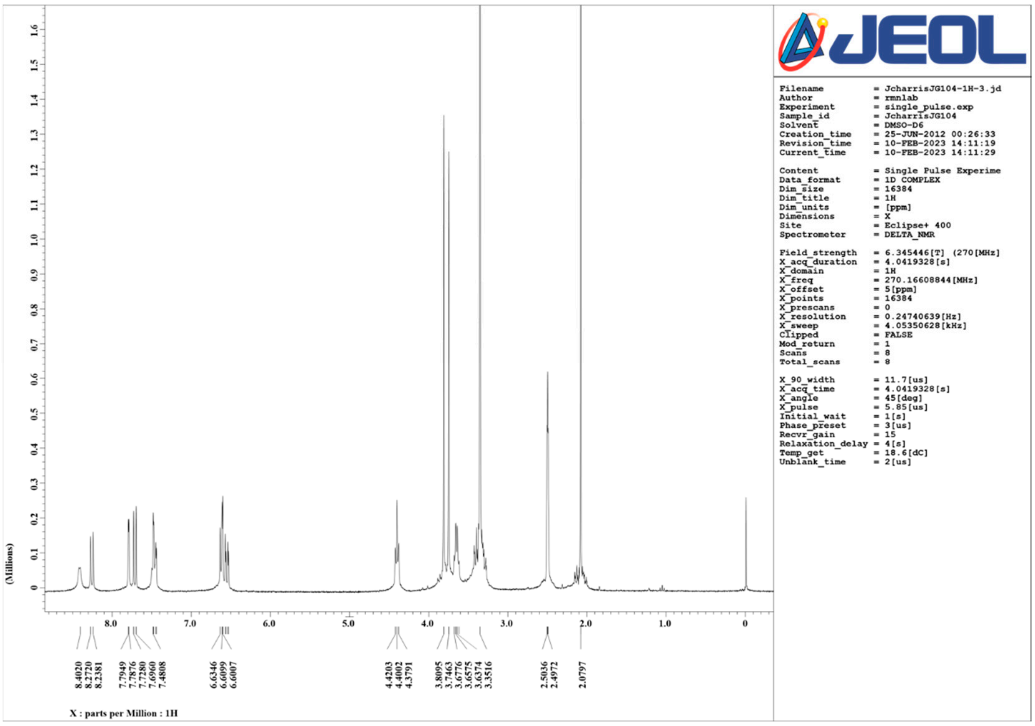

S23. Comp. 4d

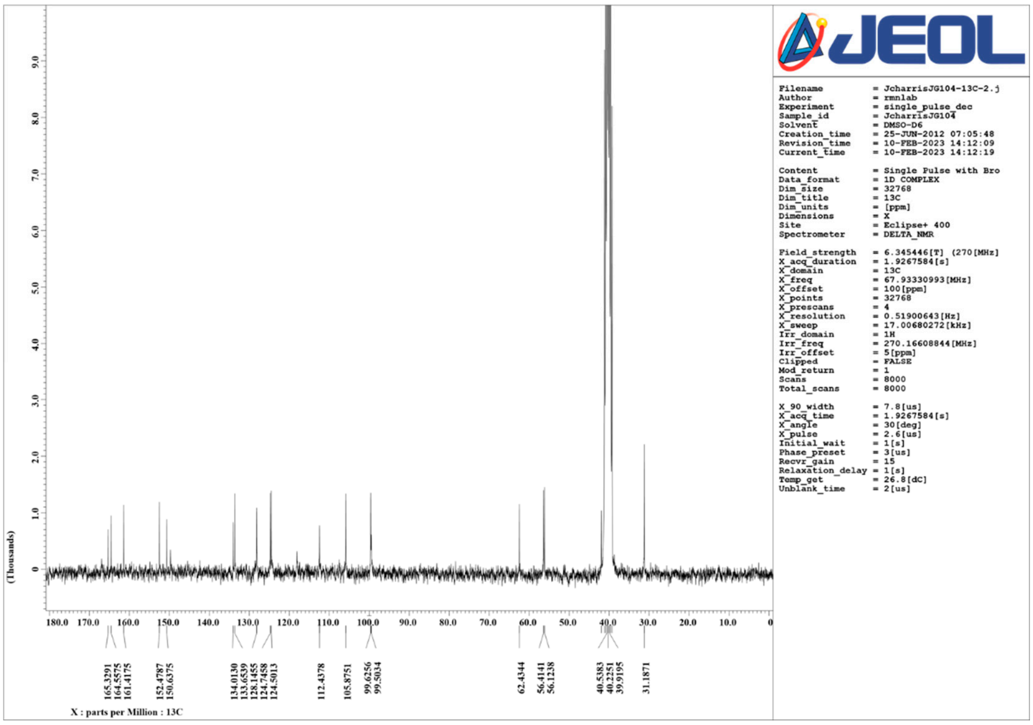
JEOL
Filename = JcharrisJG104-13C-2.j
Author = rmnlab
Experiment = single_pulse_dec
Sample_id = JcharrisJG104
Solvent = DMSO-D6
Creation_time = 25-JUN-2012 07:05:48
Revision_time = 10-FEB-2023 14:12:09
Current_time = 10-FEB-2023 14:12:19
Content = Single Pulse with Bro
Data_format = 1D COMPLEX
Dim_size = 32768
Dim_title = 13C
Dim_units = [ppm]
Dimensions = X
Site = Eclipse+ 400
Spectrometer = DELTA_NMR
Field_strength = 6.345446[T] (270[MHz]
X_acq_duration = 1.9267584[s]
X_domain = 13C
X_freq = 67.93330993[MHz]
X_offset = 100[ppm]
X_points = 32768
X_prescans = 4
X_resolution = 0.51900643[Hz]
X_sweep = 17.00680272[kHz]
Irr_domain = 1H
Irr_freq = 270.16608844[MHz]
Irr_offset = 5[ppm]
Clipped = FALSE
Mod_return = 1
Scans = 8000
Total_scans = 8000
X_90_width = 7.8[us]
X_acq_time = 1.9267584[s]
X_angle = 30[deg]
X_pulse = 2.6[us]
Initial_wait = 1[s]
Phase_preset = 3[us]
Recvr_gain = 15
Relaxation_delay = 1[s]
Temp_get = 26.8[dC]
Unblank_time = 2[us]
(Thousands)
165.3291
164.5575
161.4175
152.4787
150.6375
134.0130
133.6539
128.1455
124.7458
124.5013
112.4378
105.8751
99.6256
99.5034
62.4344
56.4141
56.1238
40.5383
40.2251
39.9195
31.1871
X : parts per Million : 13C

S24. Comp. 4e

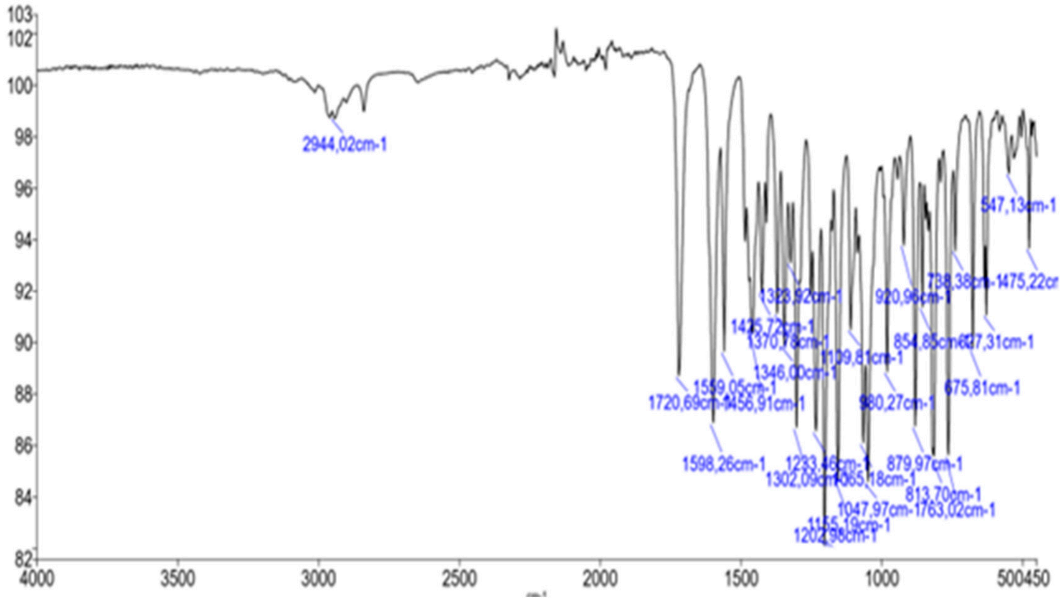

S25. Comp. 4e

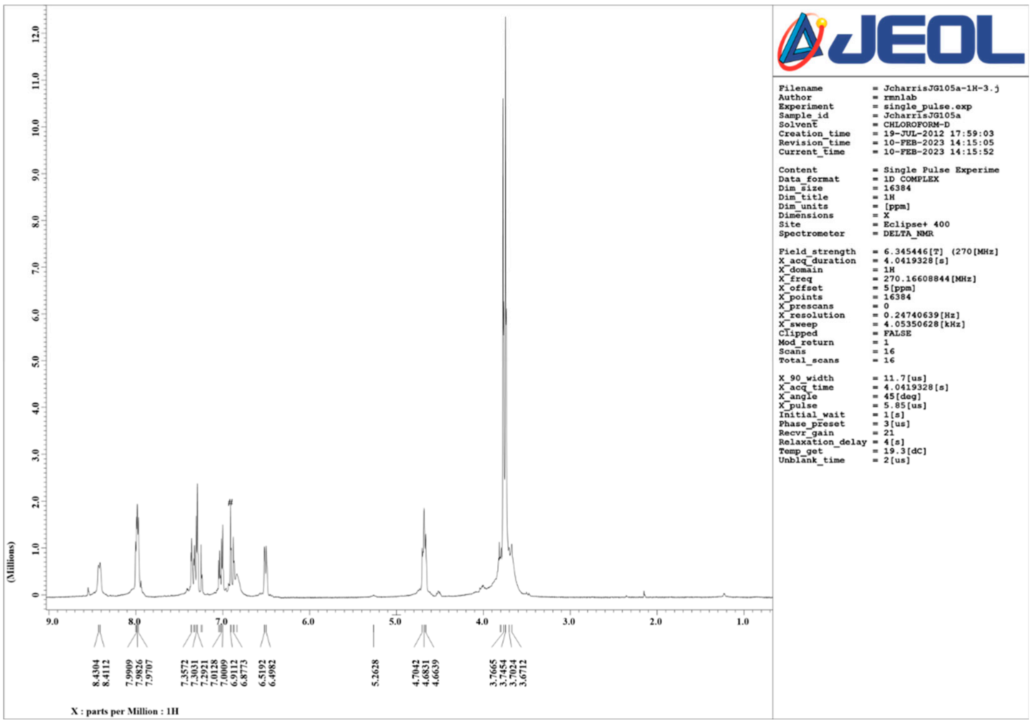

S26. Comp. 4e

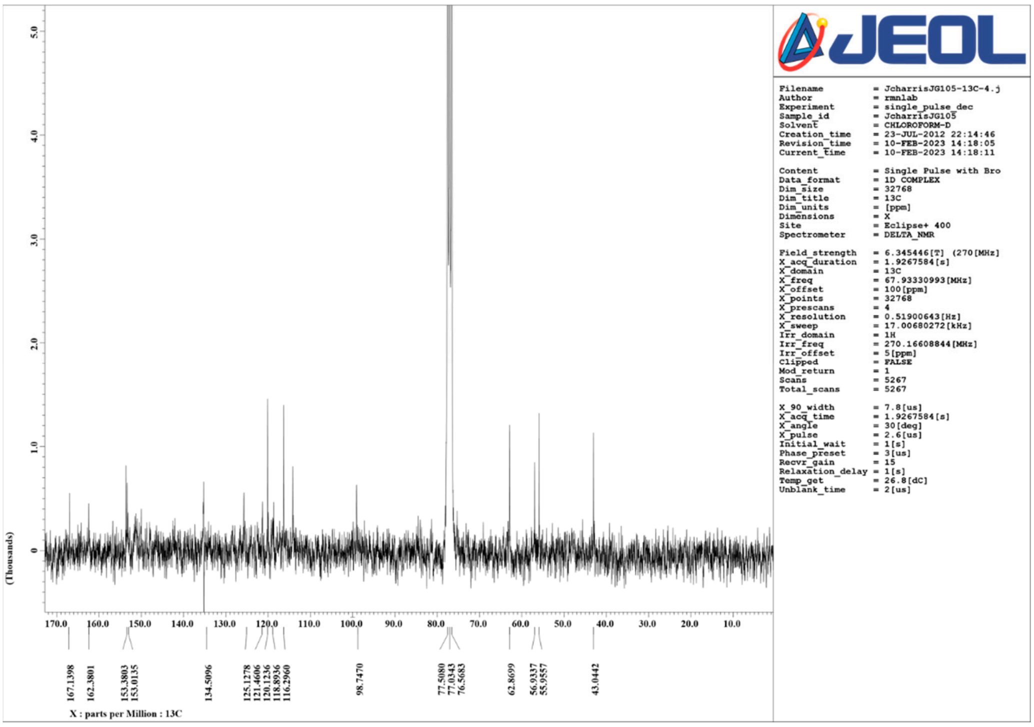

S27. Comp. 4e

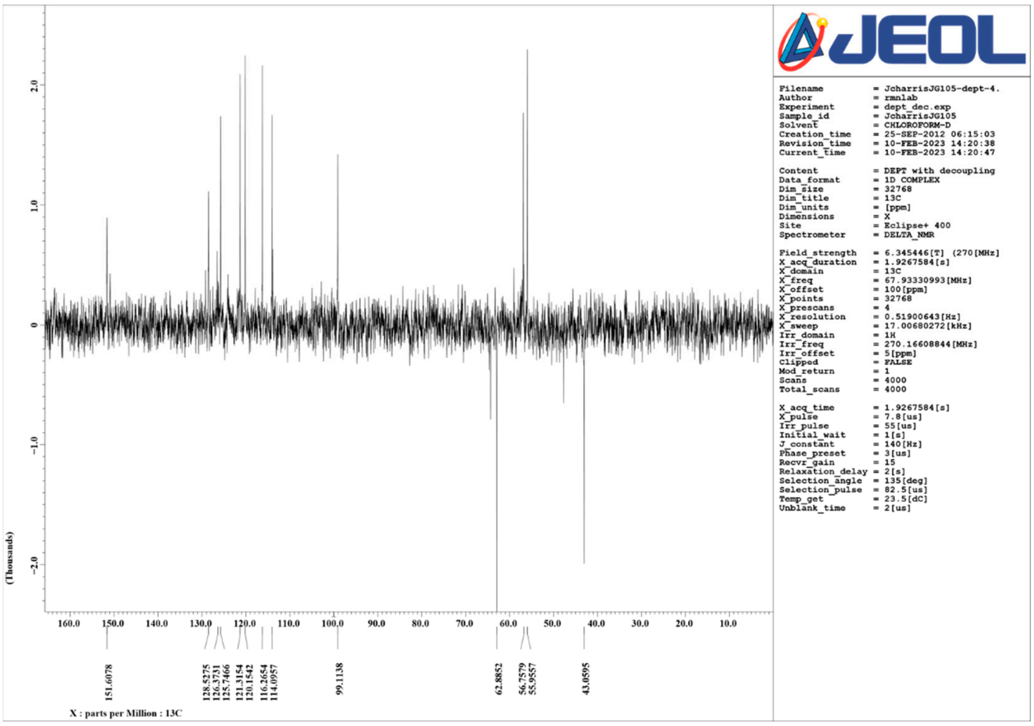

S28. Comp. 4f

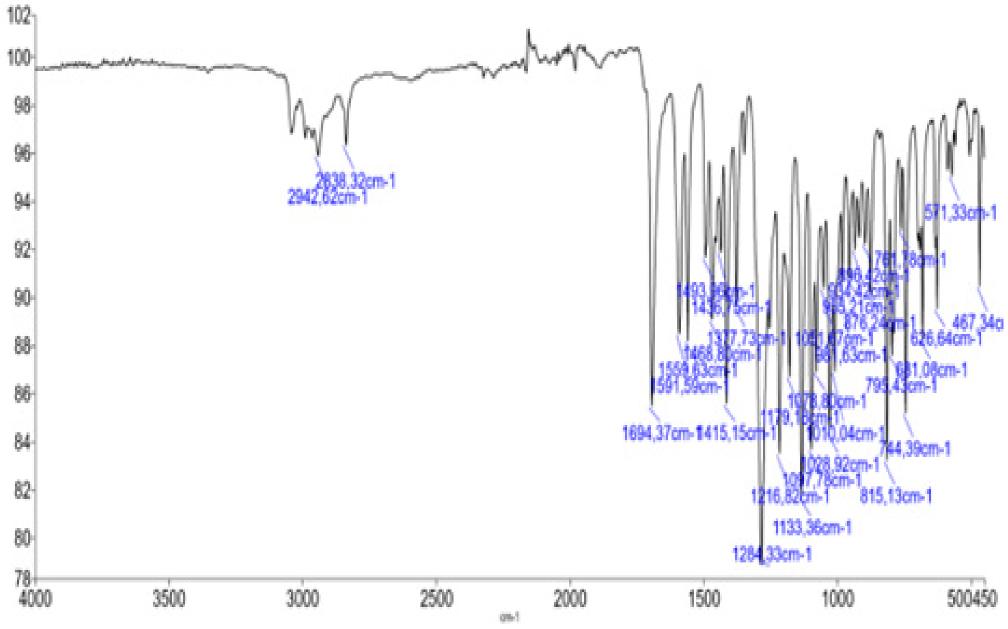

S29. Comp. 4f

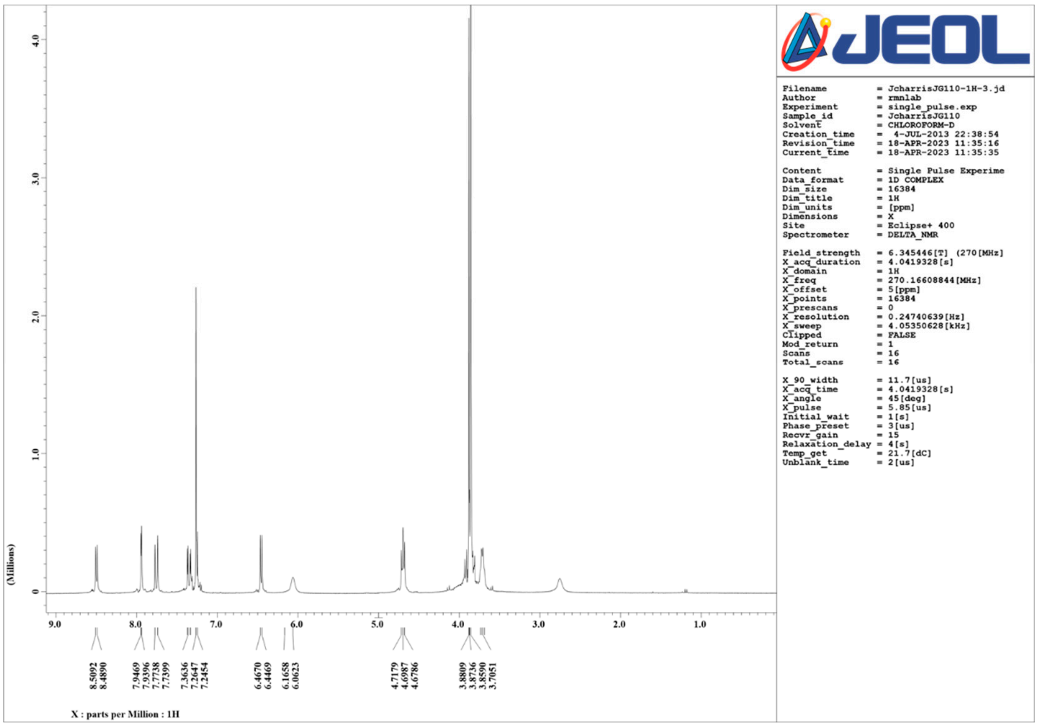

S30. Comp. 4f

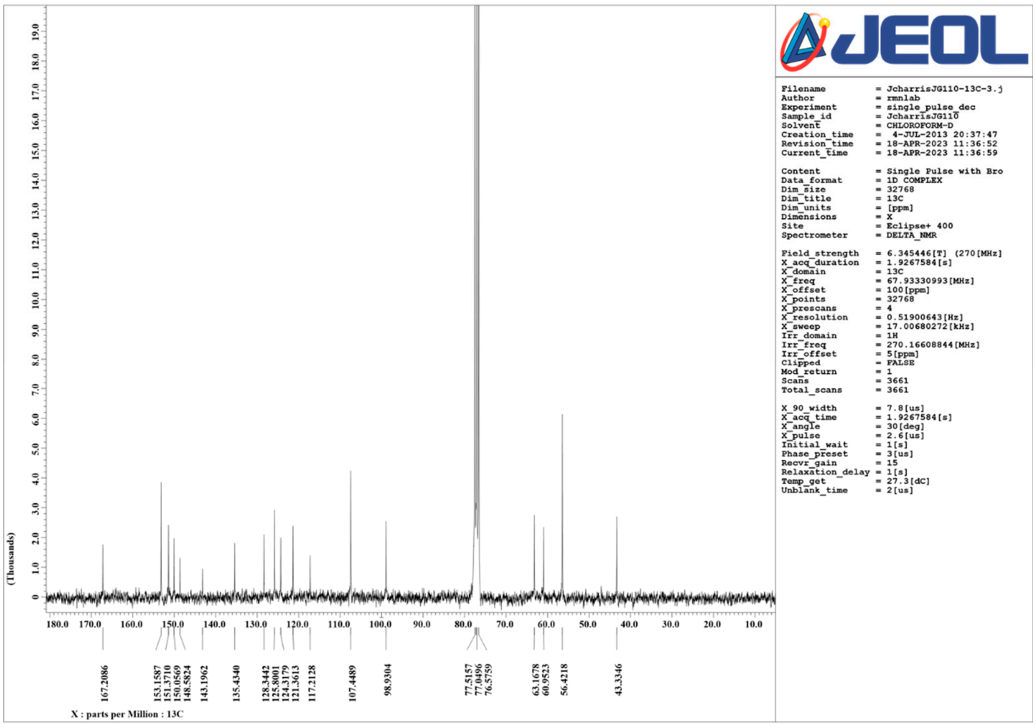

S31. Comp. 4f

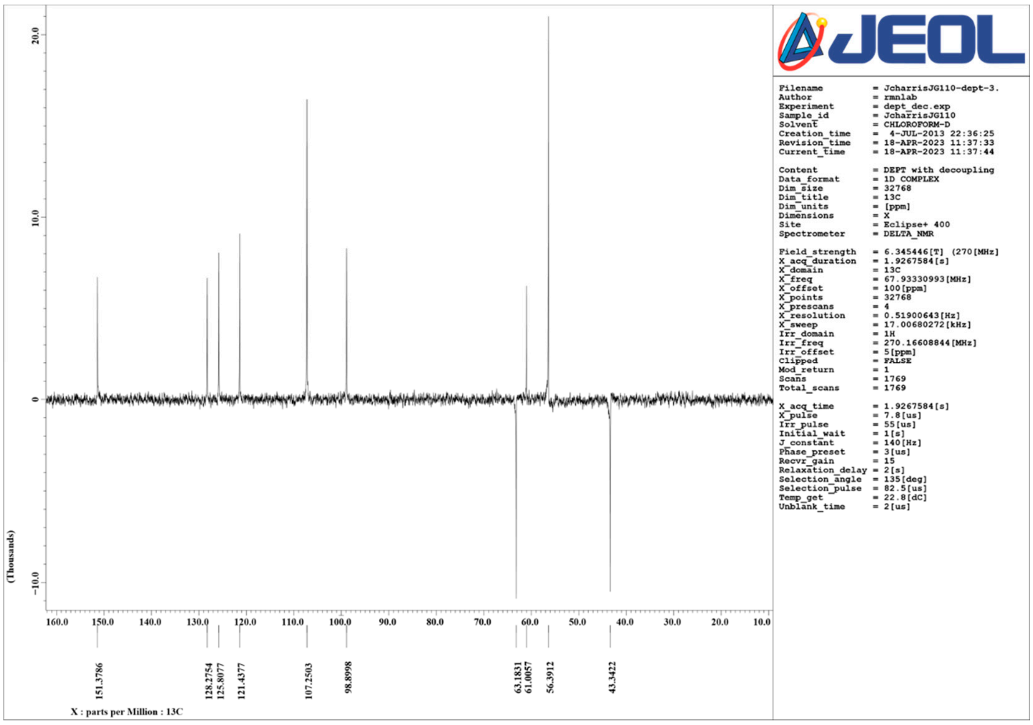

S32. Comp. 4f

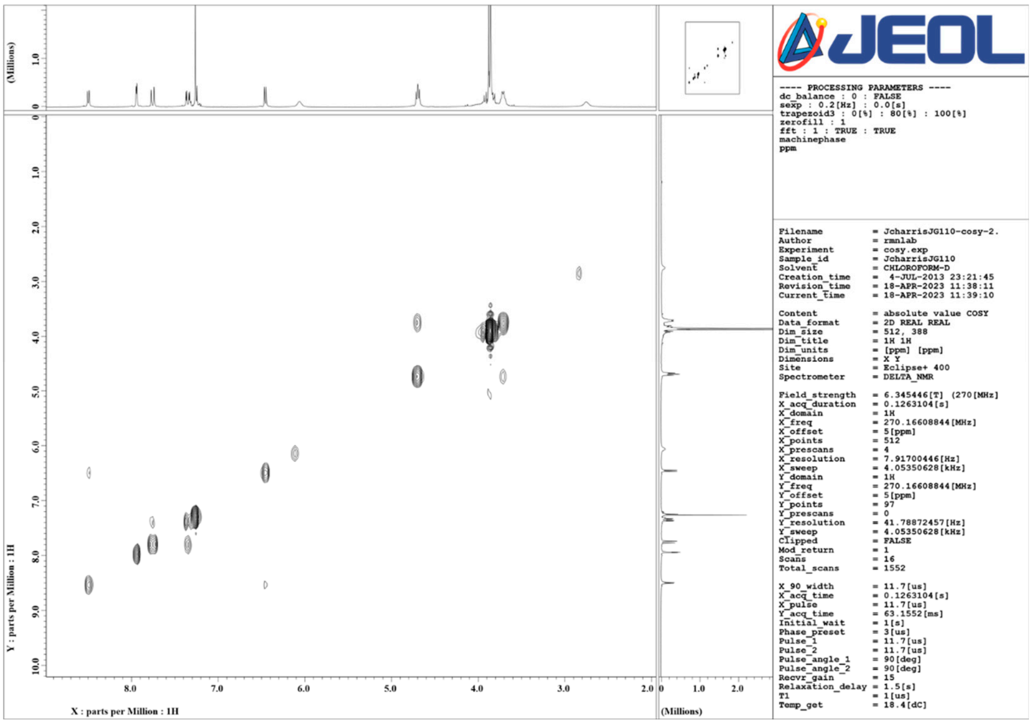

JEOL
---- PROCESSING PARAMETERS ----
dc_balance : 0 : FALSE
sexp : 0.2[Hz] : 0.0[s]
trapezoid3 : 0[%] : 80[%] : 100[%]
zerofill : 1
fft : 1 : TRUE : TRUE
machinephase
ppm
Filename = JcharrisJG110-cosy-2.
Author = rmnlab
Experiment = cosy.exp
Sample_id = JcharrisJG110
Solvent = CHLOROFORM-D
Creation_time = 4-JUL-2013 23:21:45
Revision_time = 18-APR-2023 11:38:11
Current_time = 18-APR-2023 11:39:10
Content = absolute value COSY
Data_format = 2D REAL REAL
Dim_size = 512, 388
Dim_title = 1H 1H
Dim_units = [ppm] [ppm]
Dimensions = X Y
Site = Eclipse+ 400
Spectrometer = DELTA_NMR
Field_strength = 6.345446[T] (270[MHz]
X_acq_duration = 0.1263104[s]
X_domain = 1H
X_freq = 270.16608844[MHz]
X_offset = 5[ppm]
X_points = 512
X_prescans = 4
X_resolution = 7.91700446[Hz]
X_sweep = 4.05350628[kHz]
Y_domain = 1H
Y_freq = 270.16608844[MHz]
Y_offset = 5[ppm]
Y_points = 97
Y_prescans = 0
Y_resolution = 41.78872457[Hz]
Y_sweep = 4.05350628[kHz]
Clipped = FALSE
Mod_return = 1
Scans = 16
Total_scans = 1552
X_90_width = 11.7[us]
X_acq_time = 0.1263104[s]
X_pulse = 11.7[us]
Y_acq_time = 63.1552[ms]
Initial_wait = 1[s]
Phase_preset = 3[us]
Pulse_1 = 11.7[us]
Pulse_2 = 11.7[us]
Pulse_angle_1 = 90[deg]
Pulse_angle_2 = 90[deg]
Recvr_gain = 15
Relaxation_delay = 1.5[s]
T1 = 1[us]
Temp_get = 18.4[dC]
X : parts per Million : 1H
Y : parts per Million : 1H
(Millions)

S33. Comp. 4g

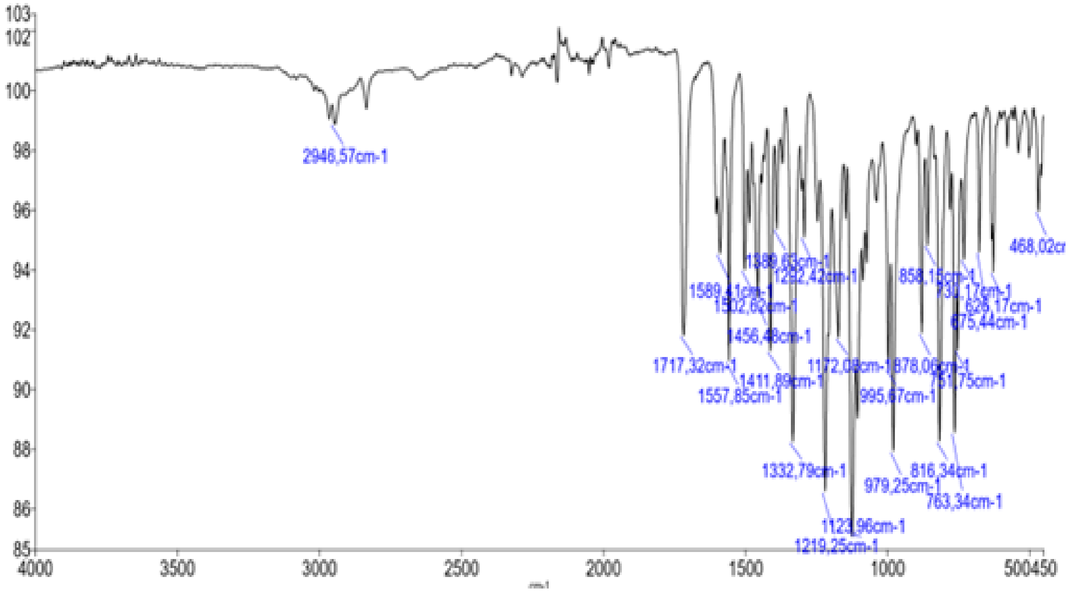

S34. Comp. 4g

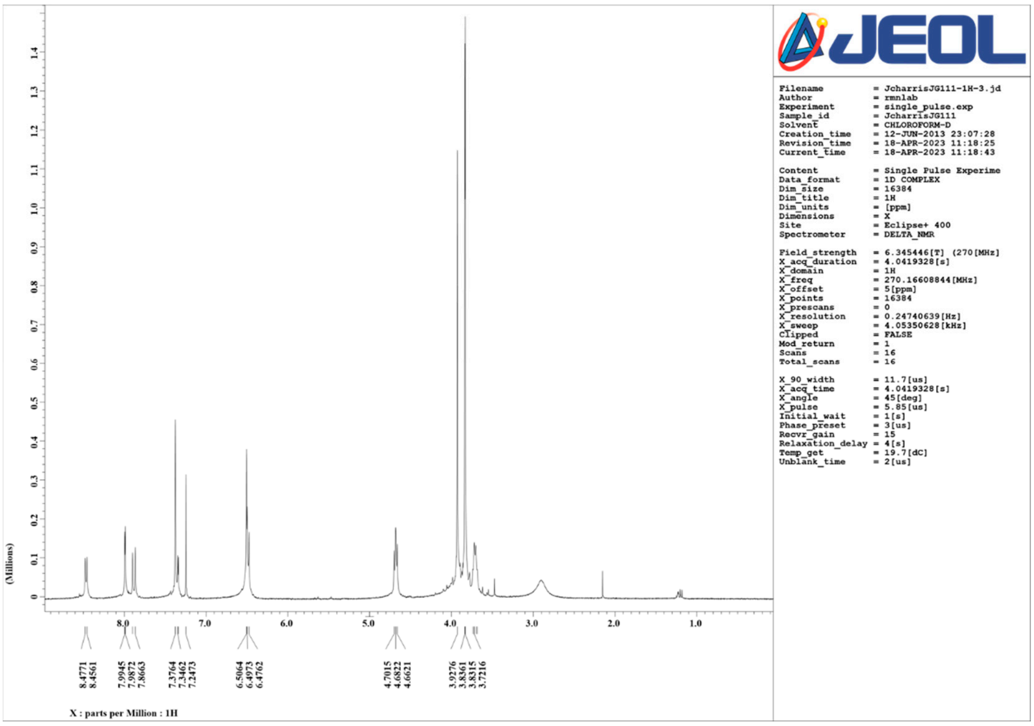

S35. Comp. 4g

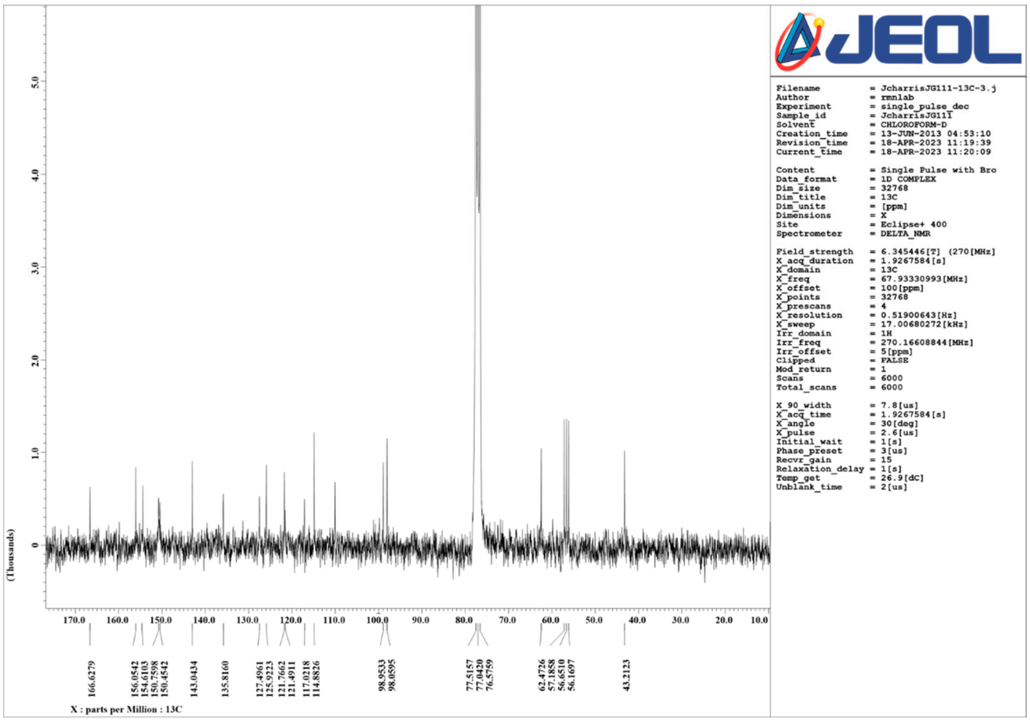

S36. Comp. 4g

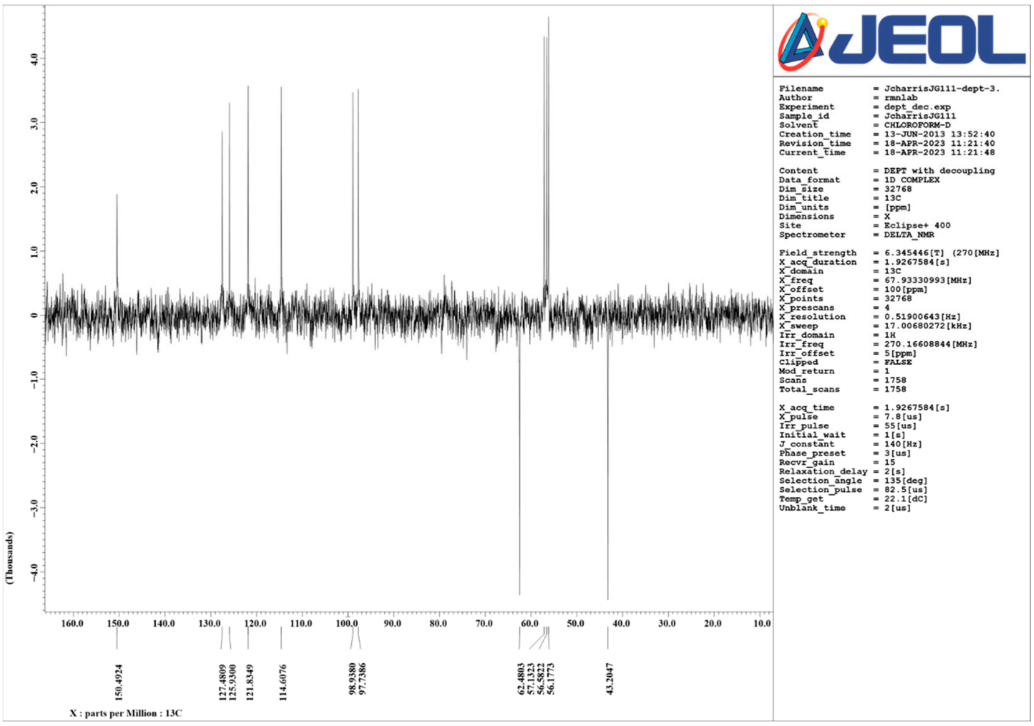

S37. Comp. 4g

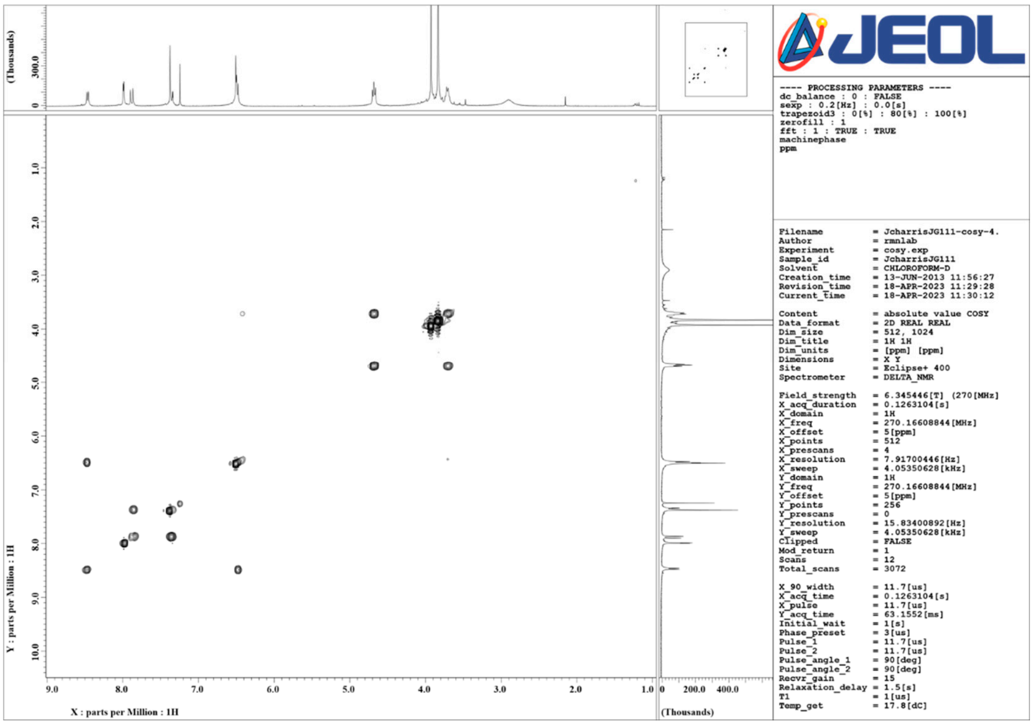

S38. Comp. 4g

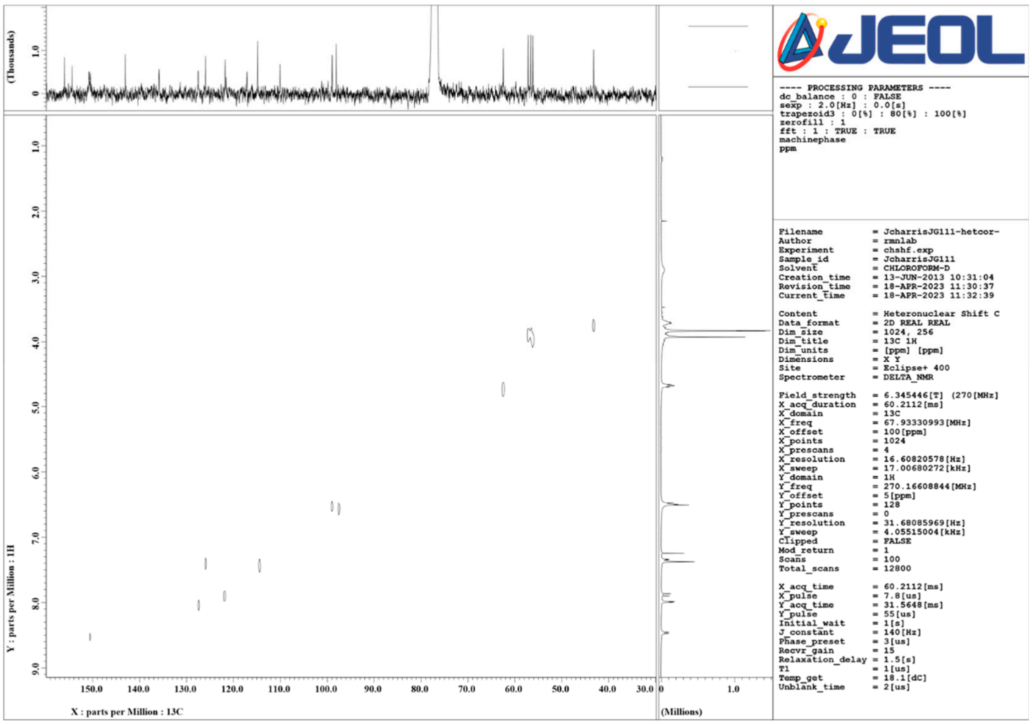

S39. Comp. 4h

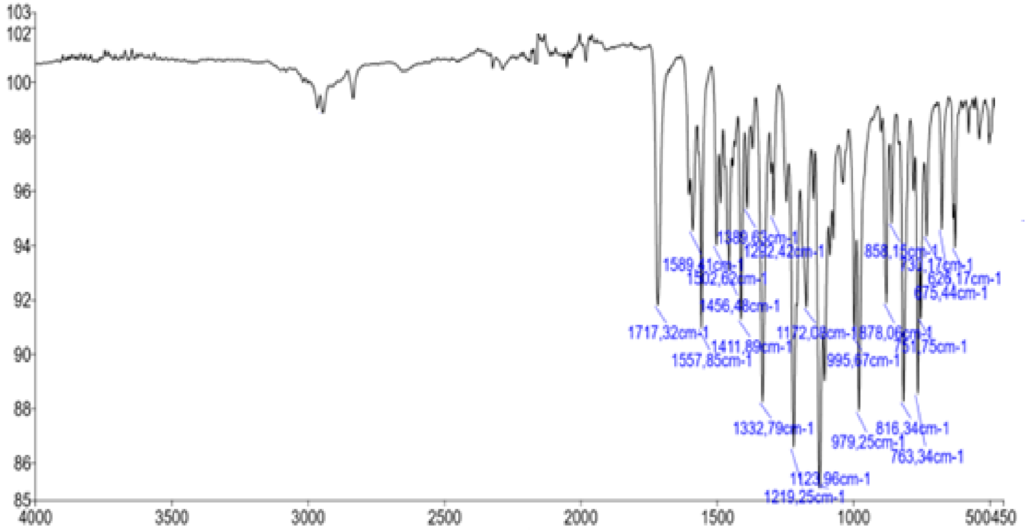

S40. Comp. 4h

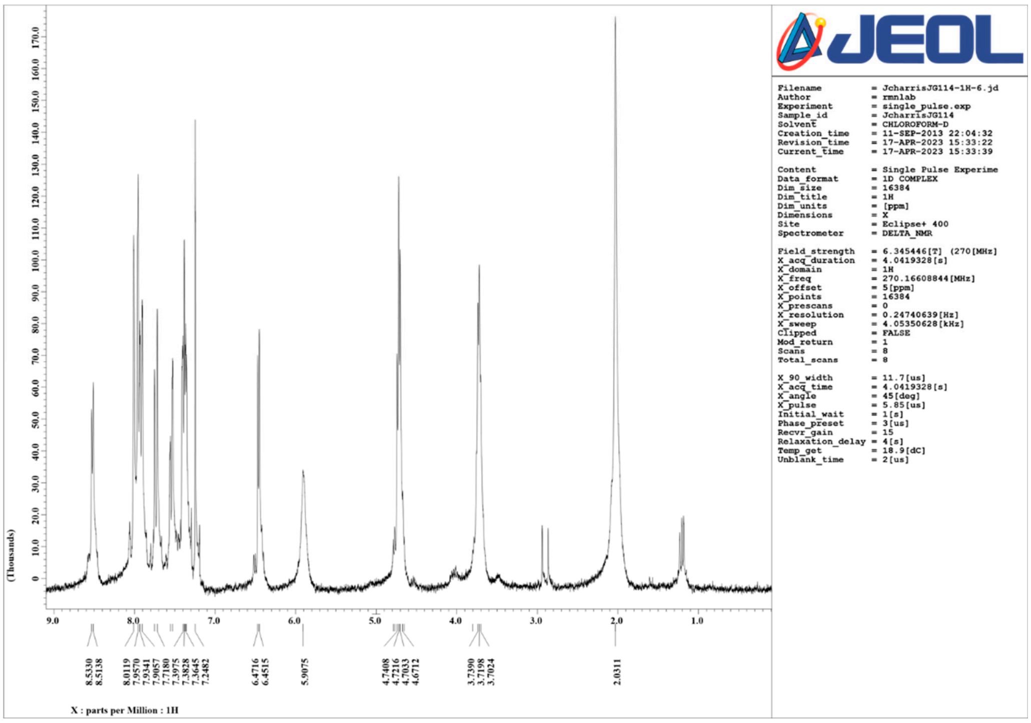

S41. Comp. 4h

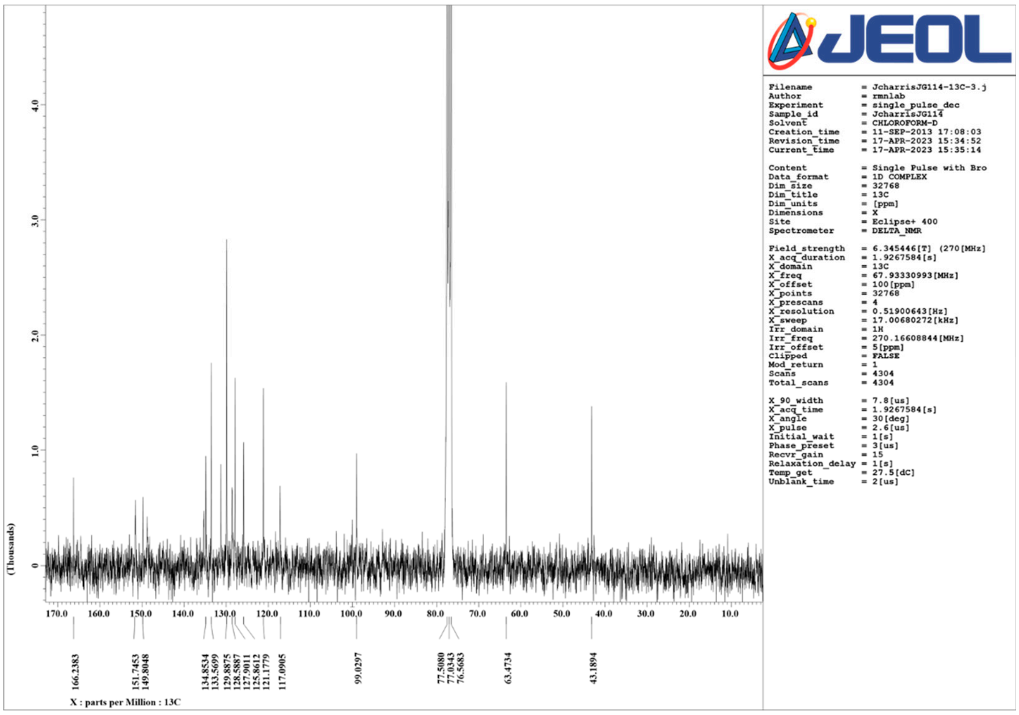

S42. Comp. 4h

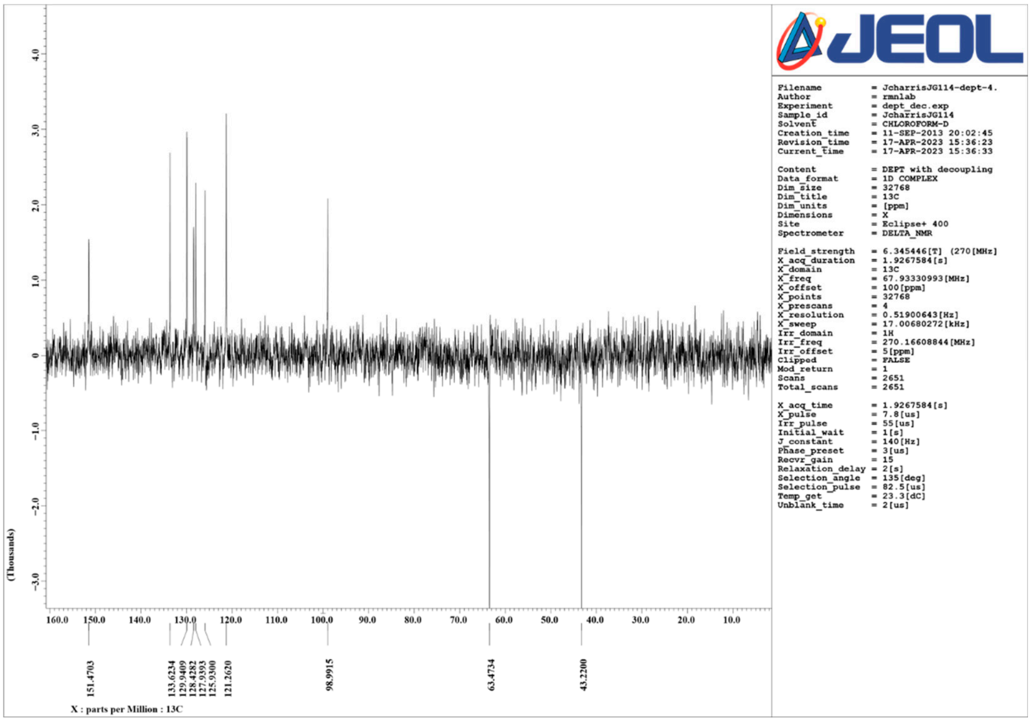

S43. Comp. 4h

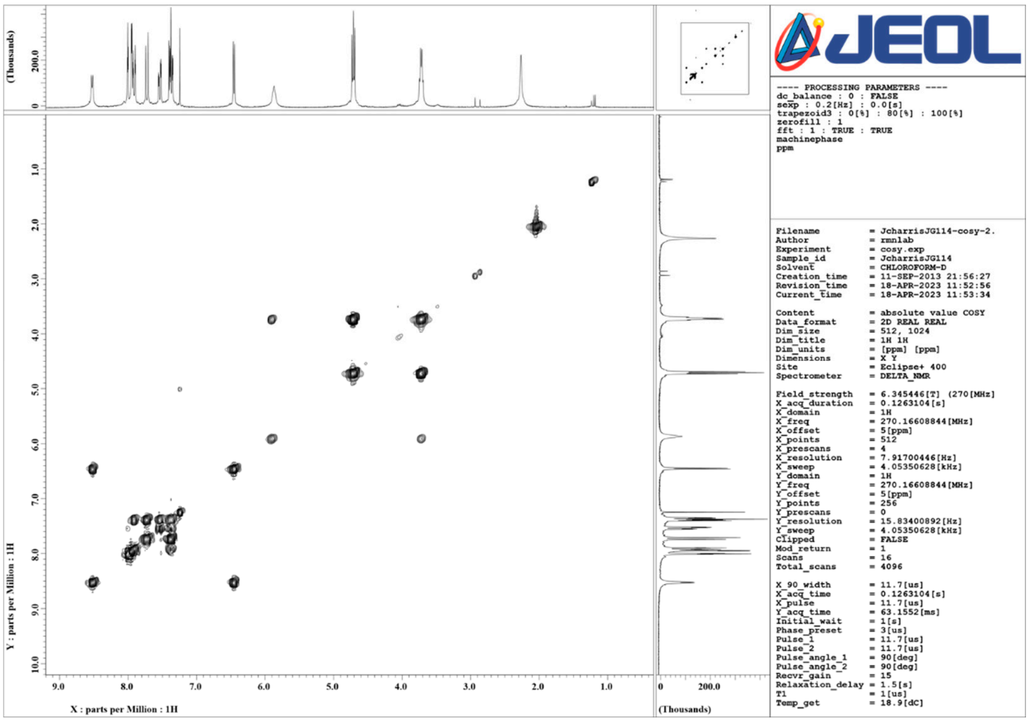
(Thousands)
200.0
1.0
2.0
3.0
4.0
5.0
6.0
7.0
8.0
9.0
10.0
Y : parts per Million : 1H
9.0
8.0
7.0
6.0
5.0
4.0
3.0
2.0
1.0
X : parts per Million : 1H
0
200.0
(Thousands)
JEOL
---- PROCESSING PARAMETERS ----
dc_balance : 0 : FALSE
sexp : 0.2[Hz] : 0.0[s]
trapezoid3 : 0[%] : 80[%] : 100[%]
zerofill : 1
fft : 1 : TRUE : TRUE
machinephase
ppm
Filename = JcharrisJG114-cosy-2.
Author = rmnlab
Experiment = cosy.exp
Sample_id = JcharrisJG114
Solvent = CHLOROFORM-D
Creation_time = 11-SEP-2013 21:56:27
Revision_time = 18-APR-2023 11:52:56
Current_time = 18-APR-2023 11:53:34
Content = absolute value COSY
Data_format = 2D REAL REAL
Dim_size = 512, 1024
Dim_title = 1H 1H
Dim_units = [ppm] [ppm]
Dimensions = X Y
Site = Eclipse+ 400
Spectrometer = DELTA_NMR
Field_strength = 6.345446[T] (270[MHz]
X_acq_duration = 0.1263104[s]
X_domain = 1H
X_freq = 270.16608844[MHz]
X_offset = 5[ppm]
X_points = 512
X_prescans = 4
X_resolution = 7.91700446[Hz]
X_sweep = 4.05350628[kHz]
Y_domain = 1H
Y_freq = 270.16608844[MHz]
Y_offset = 5[ppm]
Y_points = 256
Y_prescans = 0
Y_resolution = 15.83400892[Hz]
Y_sweep = 4.05350628[kHz]
Clipped = FALSE
Mod_return = 1
Scans = 16
Total_scans = 4096
X_90_width = 11.7[us]
X_acq_time = 0.1263104[s]
X_pulse = 11.7[us]
Y_acq_time = 63.1552[ms]
Initial_wait = 1[s]
Phase_preset = 3[us]
Pulse_1 = 11.7[us]
Pulse_2 = 11.7[us]
Pulse_angle_1 = 90[deg]
Pulse_angle_2 = 90[deg]
Recvr_gain = 15
Relaxation_delay = 1.5[s]
T1 = 1[us]
Temp_get = 18.9[dC]

S44. Comp. 4i

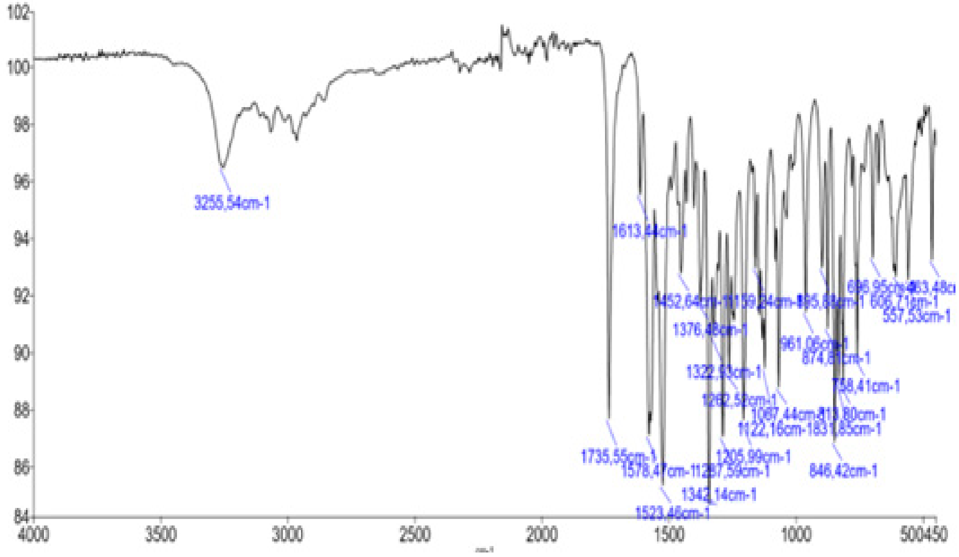

S45. Comp. 4i

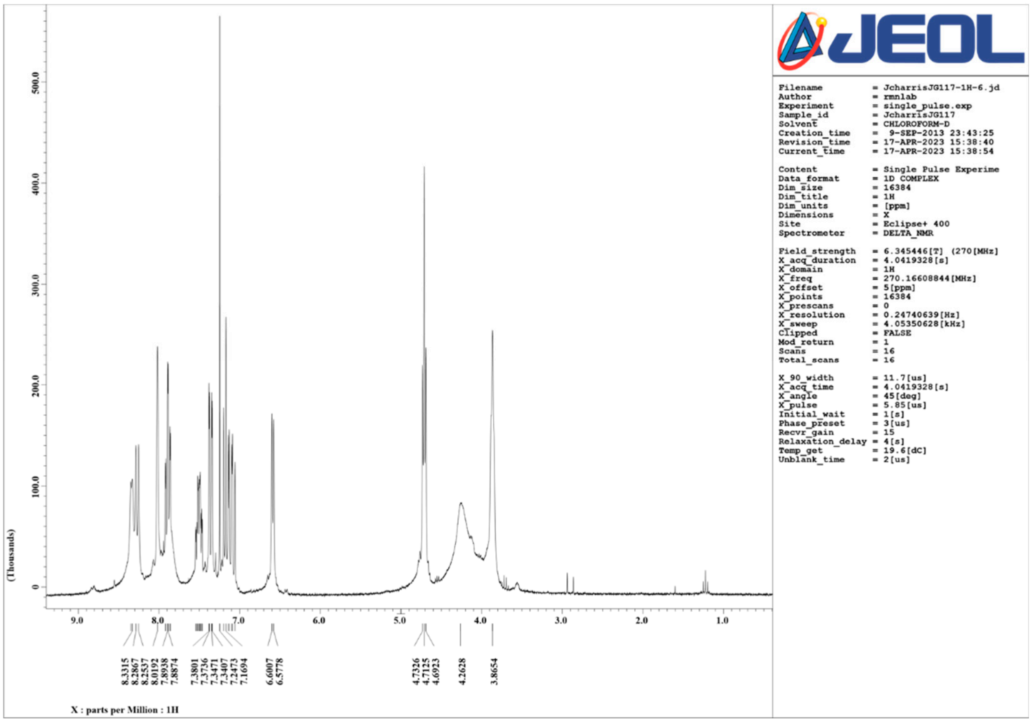

S46. Comp. 4i

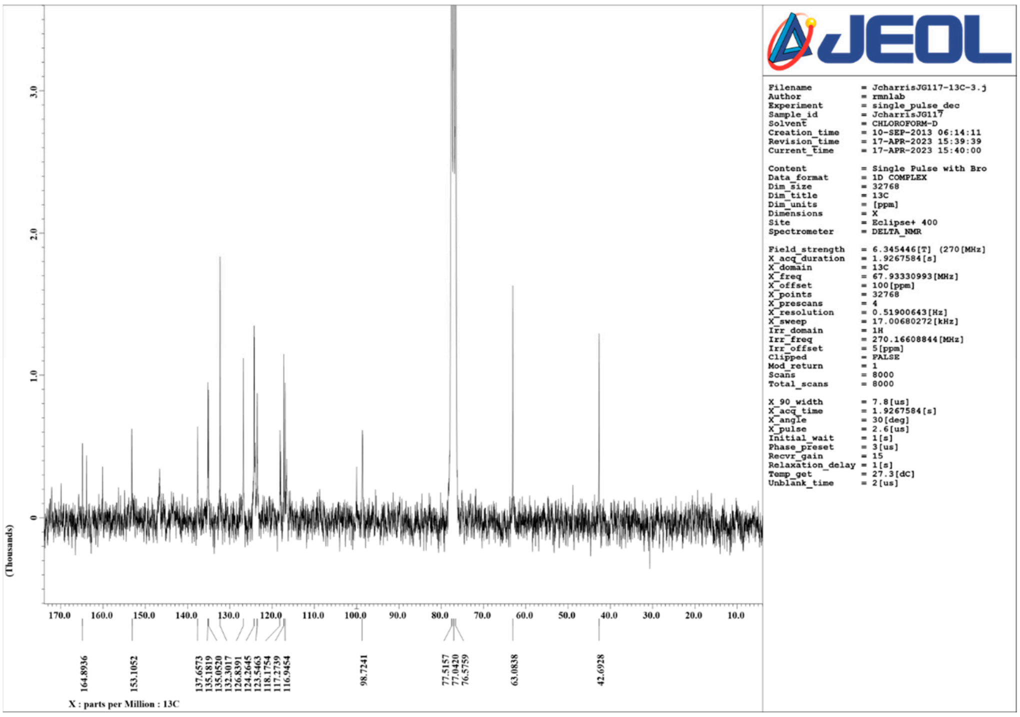

S47. Comp. 4i

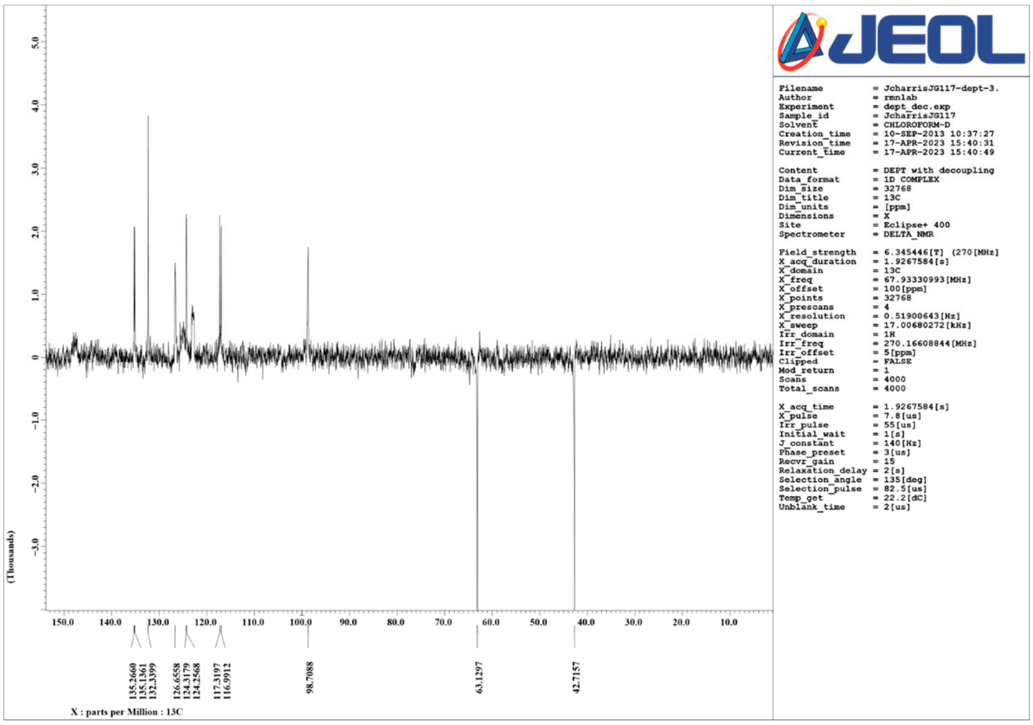

S48. Comp. 4i

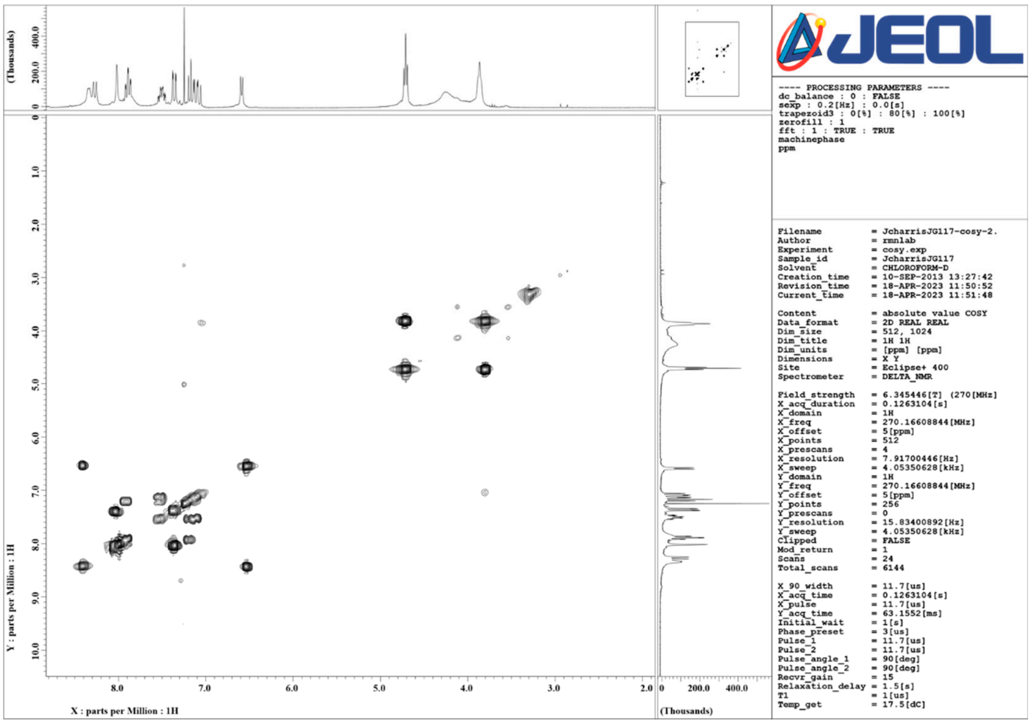
(Thousands)
X : parts per Million : 1H
Y : parts per Million : 1H
---- PROCESSING PARAMETERS ----
dc_balance : 0 : FALSE
sexp : 0.2[Hz] : 0.0[s]
trapezoid3 : 0[%] : 80[%] : 100[%]
zerofill : 1
fft : 1 : TRUE : TRUE
machinephase
ppm
Filename = JcharrisJG117-cosy-2.
Author = rmnlab
Experiment = cosy.exp
Sample_id = JcharrisJG117
Solvent = CHLOROFORM-D
Creation_time = 10-SEP-2013 13:27:42
Revision_time = 18-APR-2023 11:50:52
Current_time = 18-APR-2023 11:51:48
Content = absolute value COSY
Data_format = 2D REAL REAL
Dim_size = 512, 1024
Dim_title = 1H 1H
Dim_units = [ppm] [ppm]
Dimensions = X Y
Site = Eclipse+ 400
Spectrometer = DELTA_NMR
Field_strength = 6.345446[T] (270[MHz]
X_acq_duration = 0.1263104[s]
X_domain = 1H
X_freq = 270.16608844[MHz]
X_offset = 5[ppm]
X_points = 512
X_prescans = 4
X_resolution = 7.91700446[Hz]
X_sweep = 4.05350628[kHz]
Y_domain = 1H
Y_freq = 270.16608844[MHz]
Y_offset = 5[ppm]
Y_points = 256
Y_prescans = 0
Y_resolution = 15.83400892[Hz]
Y_sweep = 4.05350628[kHz]
Clipped = FALSE
Mod_return = 1
Scans = 24
Total_scans = 6144
X_90_width = 11.7[us]
X_acq_time = 0.1263104[s]
X_pulse = 11.7[us]
Y_acq_time = 63.1552[ms]
Initial_wait = 1[s]
Phase_preset = 3[us]
Pulse_1 = 11.7[us]
Pulse_2 = 11.7[us]
Pulse_angle_1 = 90[deg]
Pulse_angle_2 = 90[deg]
Recvr_gain = 15
Relaxation_delay = 1.5[s]
T1 = 1[us]
Temp_get = 17.5[dC]

S49. Comp. 4j

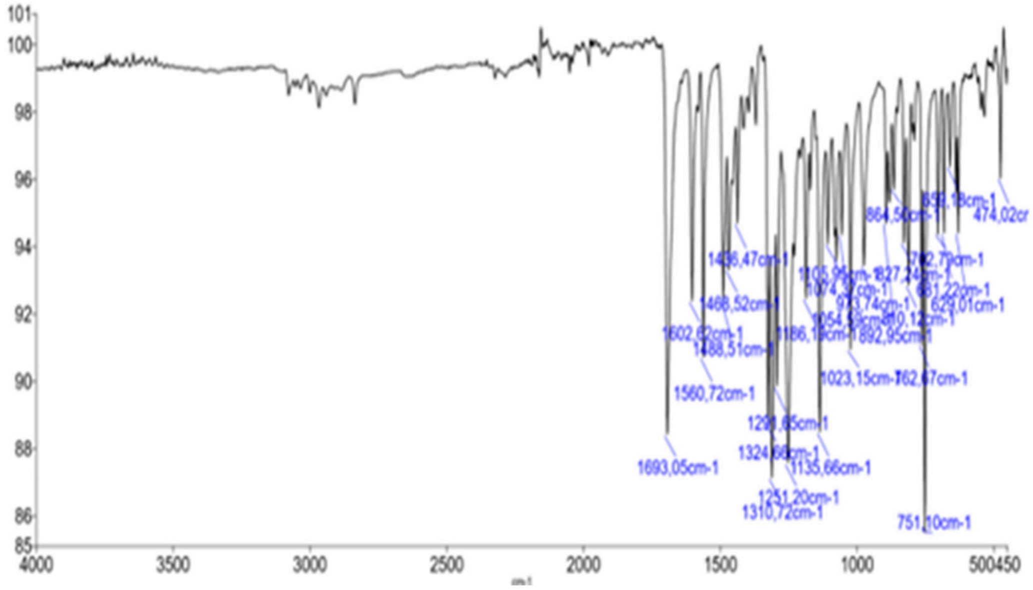

S50. Comp. 4j

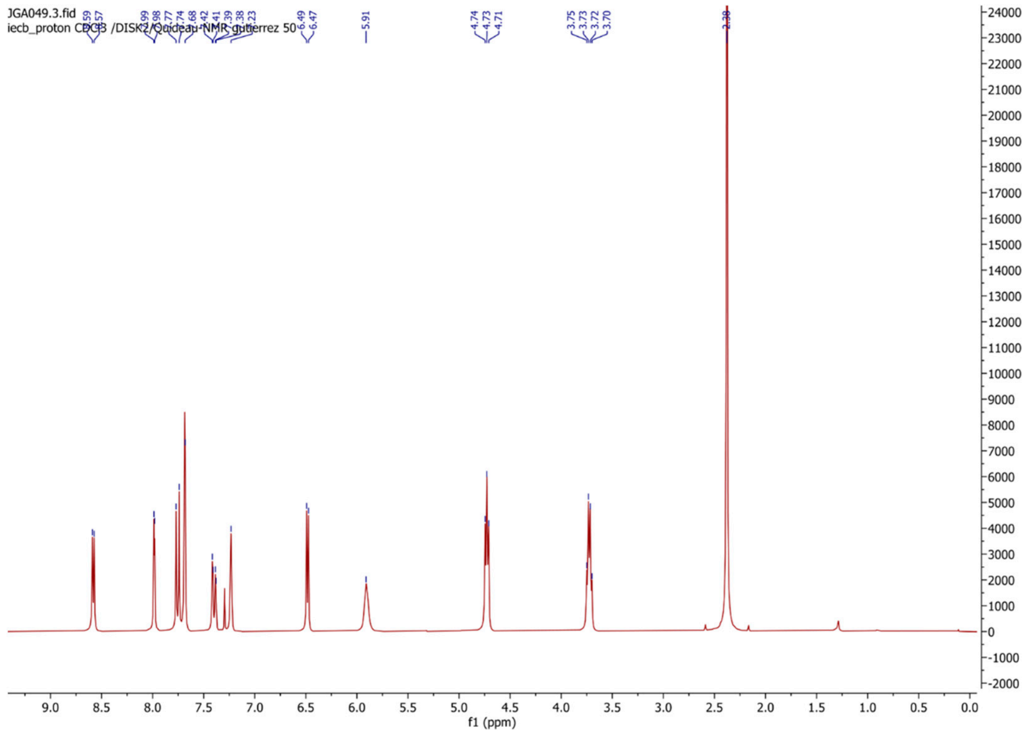

S51. Comp. 4j

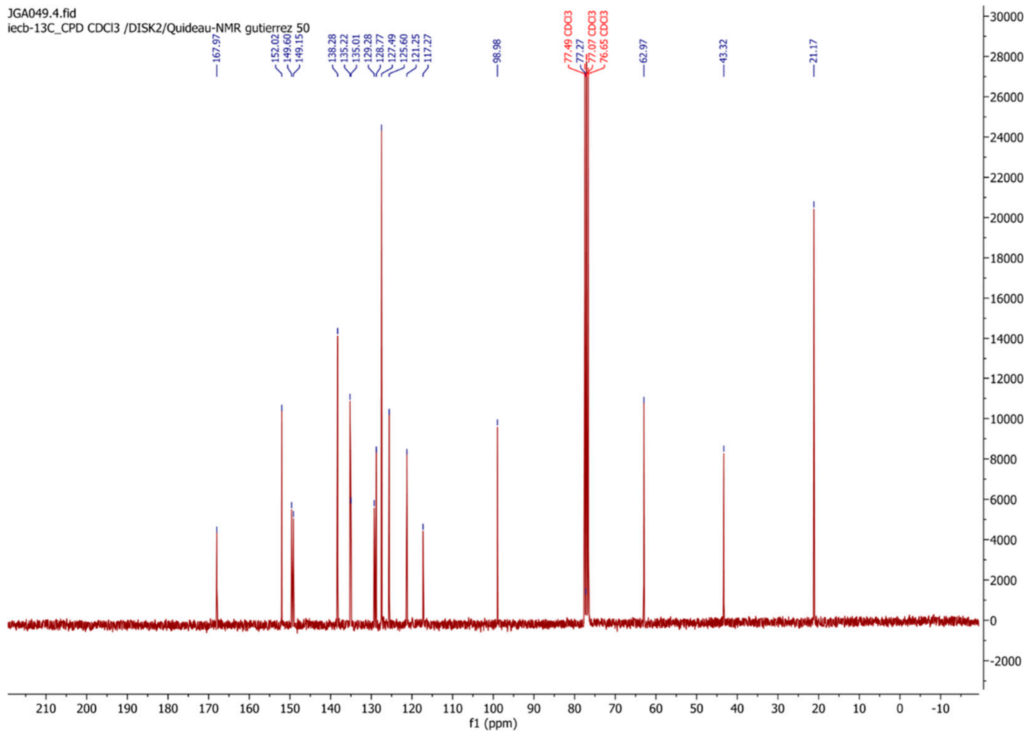

S52. Comp. 4j

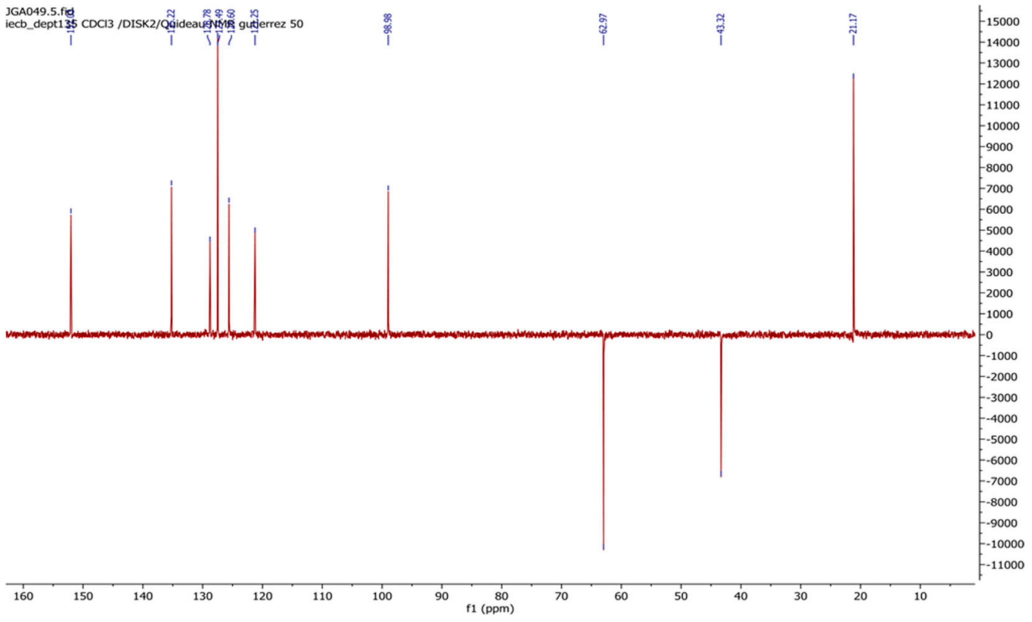

S53. Comp. 4l

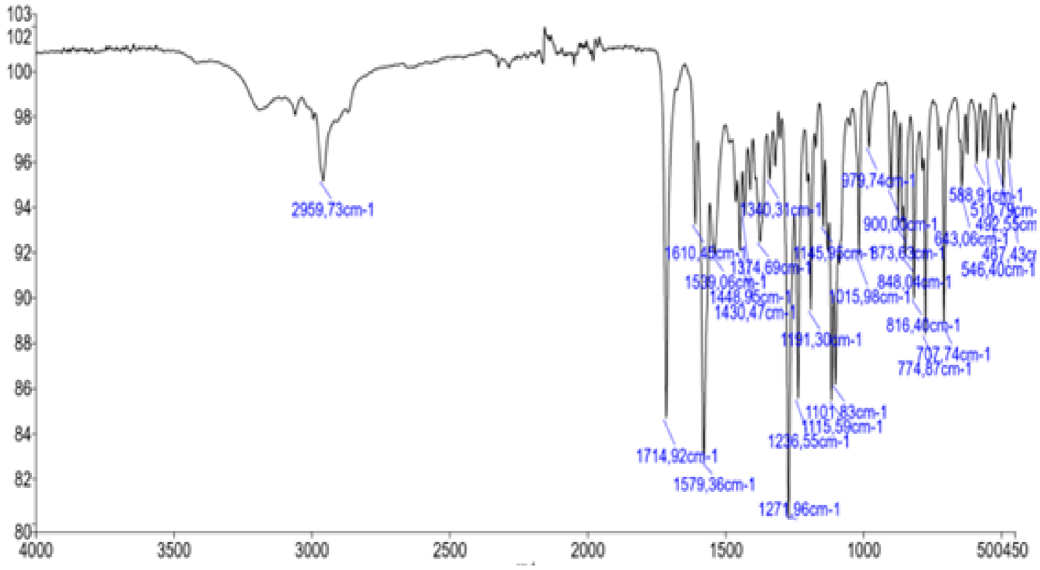

S54. Comp. 4l

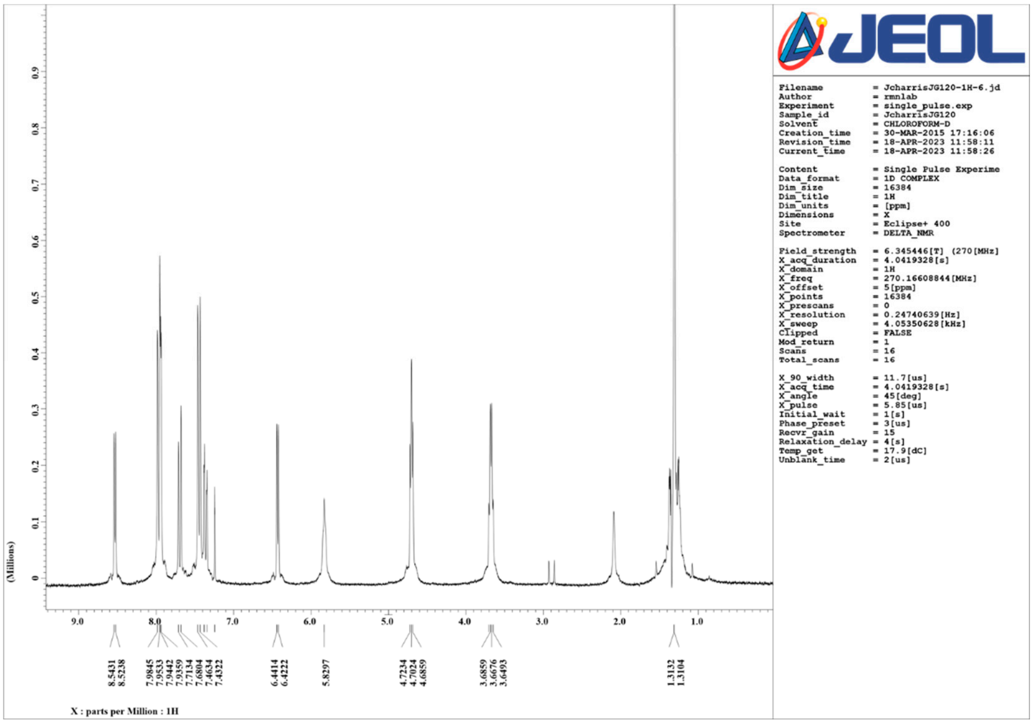

S55. Comp. 4l

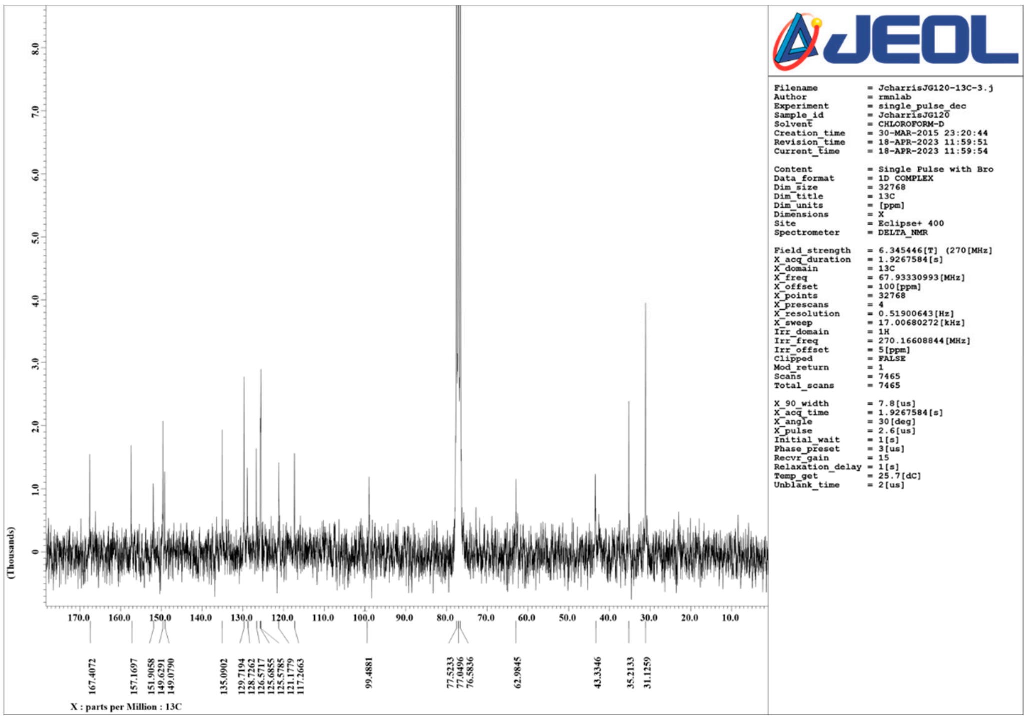

S56. Comp. 4l

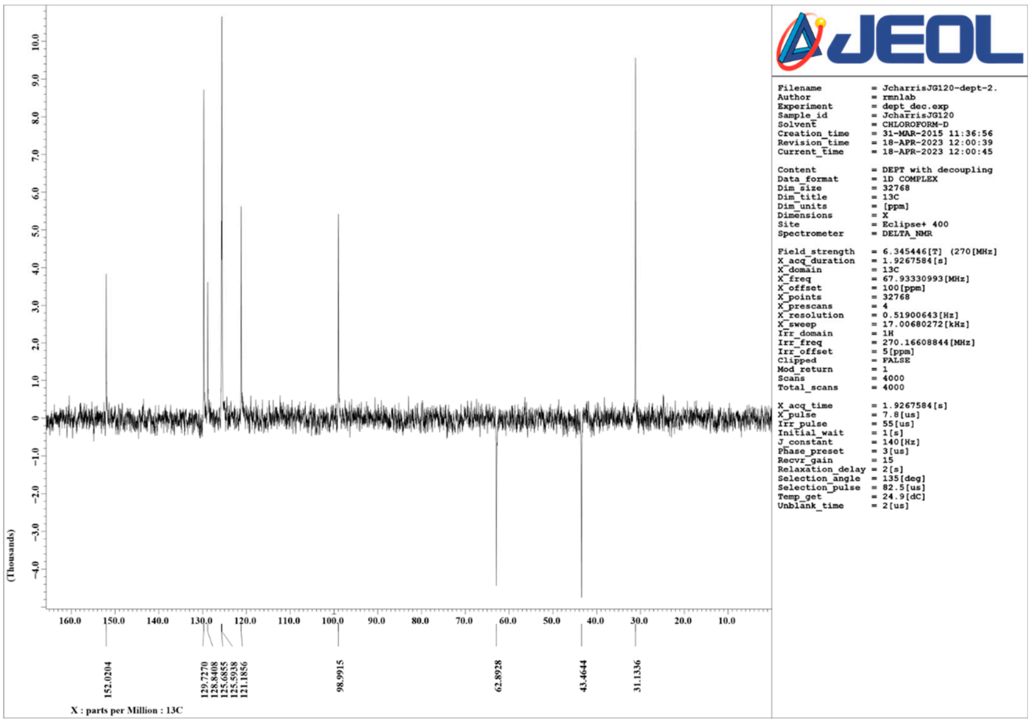

S57. Comp. 4l

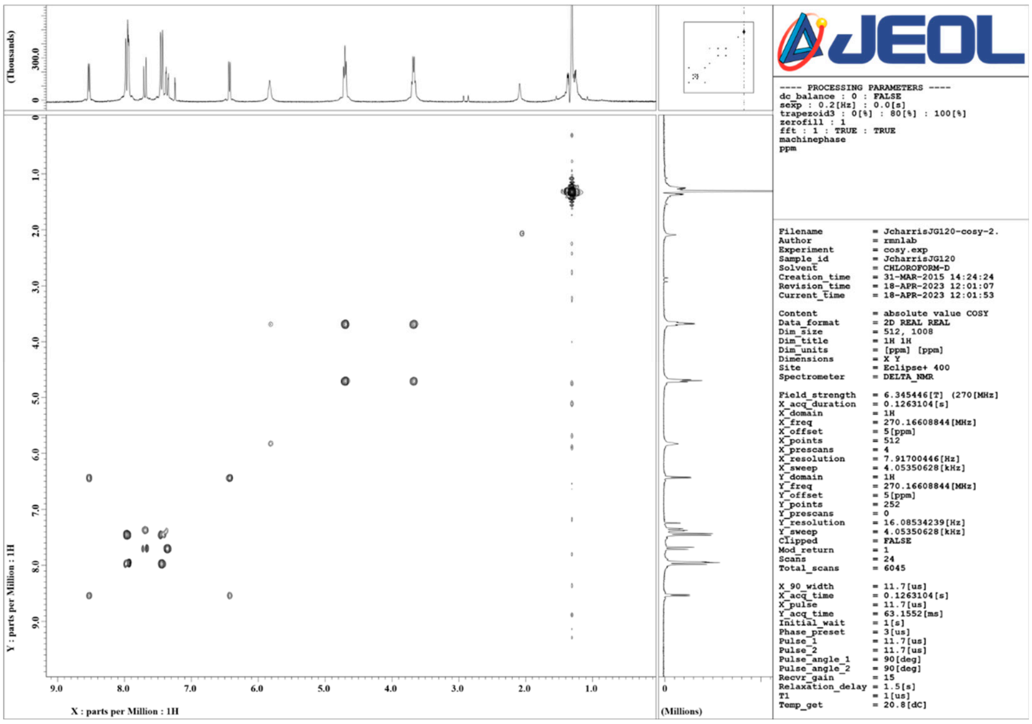

S58. Comp. 4l

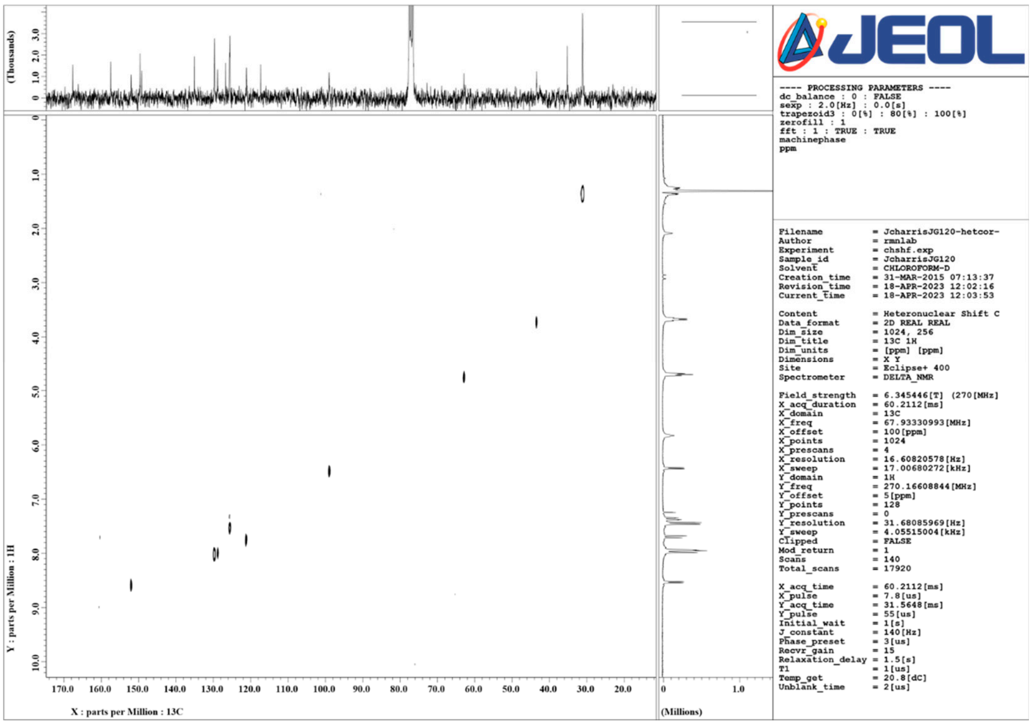

S59. Comp. 4m

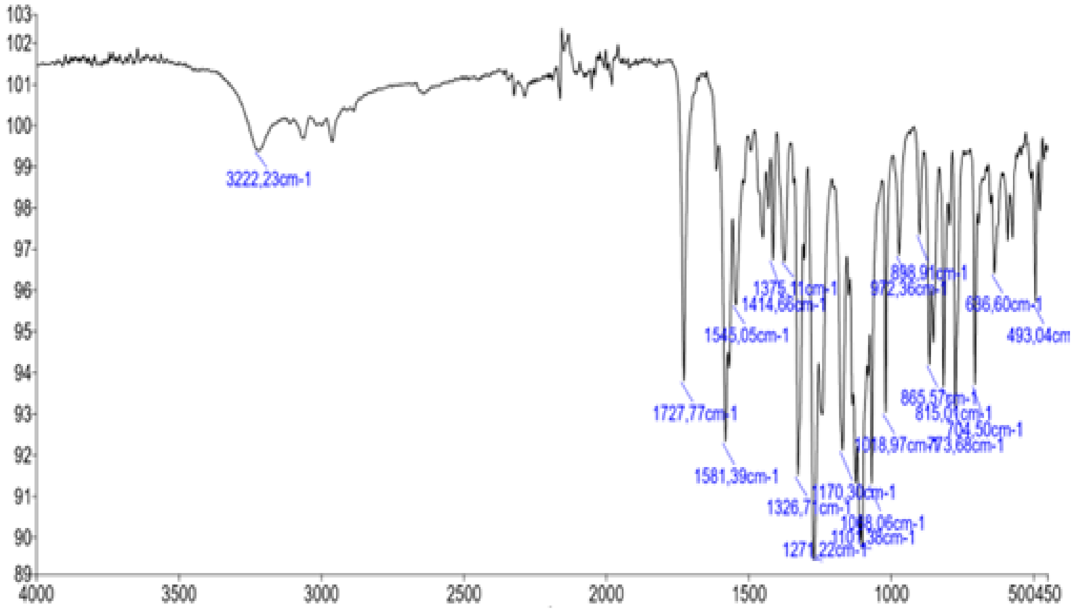

S60. Comp. 4m

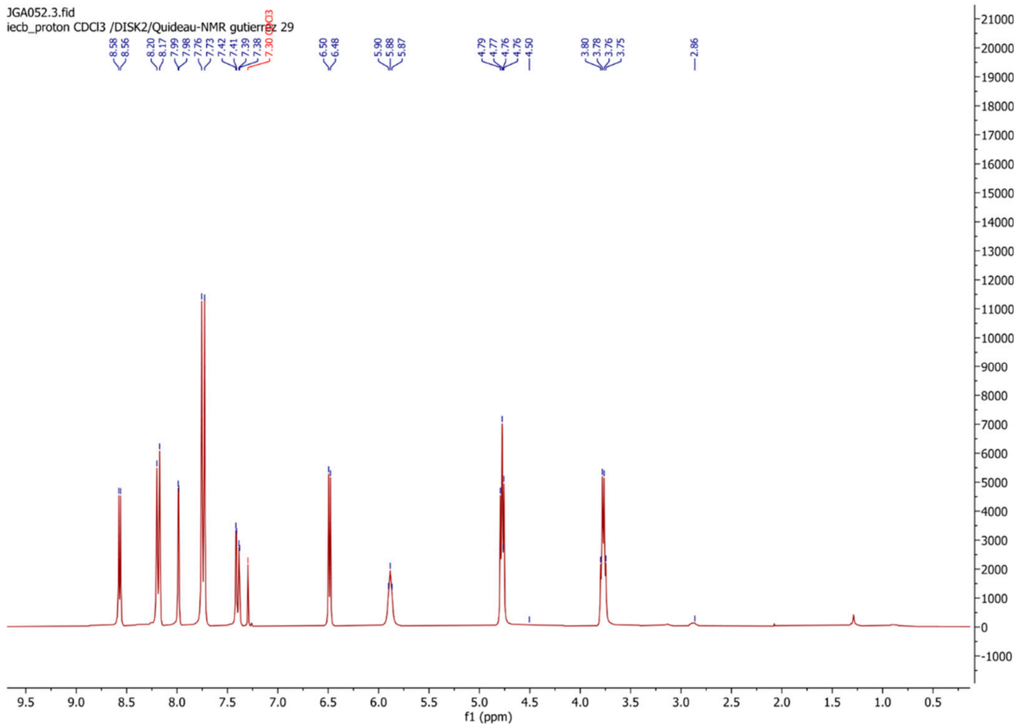

S61. Comp. 4m

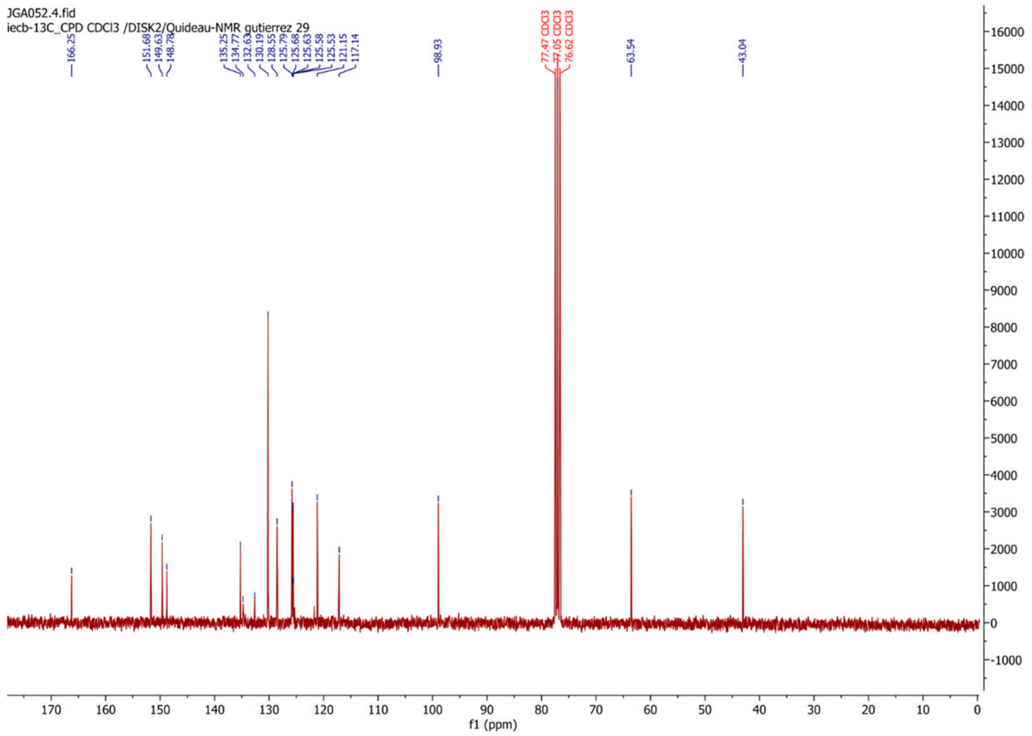

S62. Comp. 4m

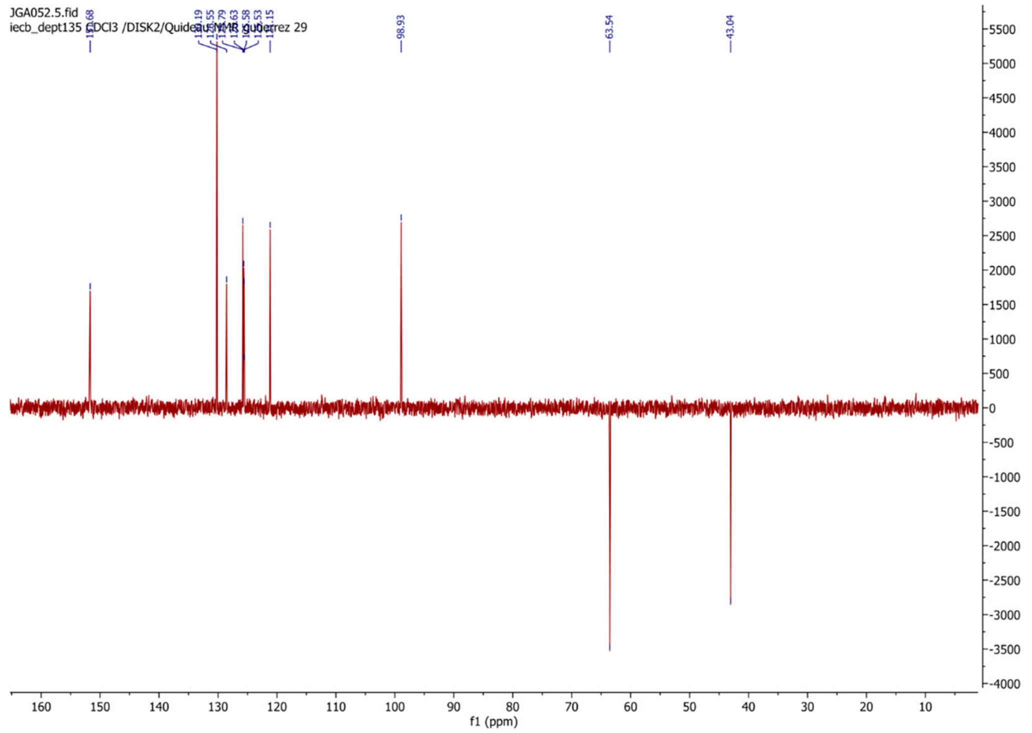

S63. Comp. 4m

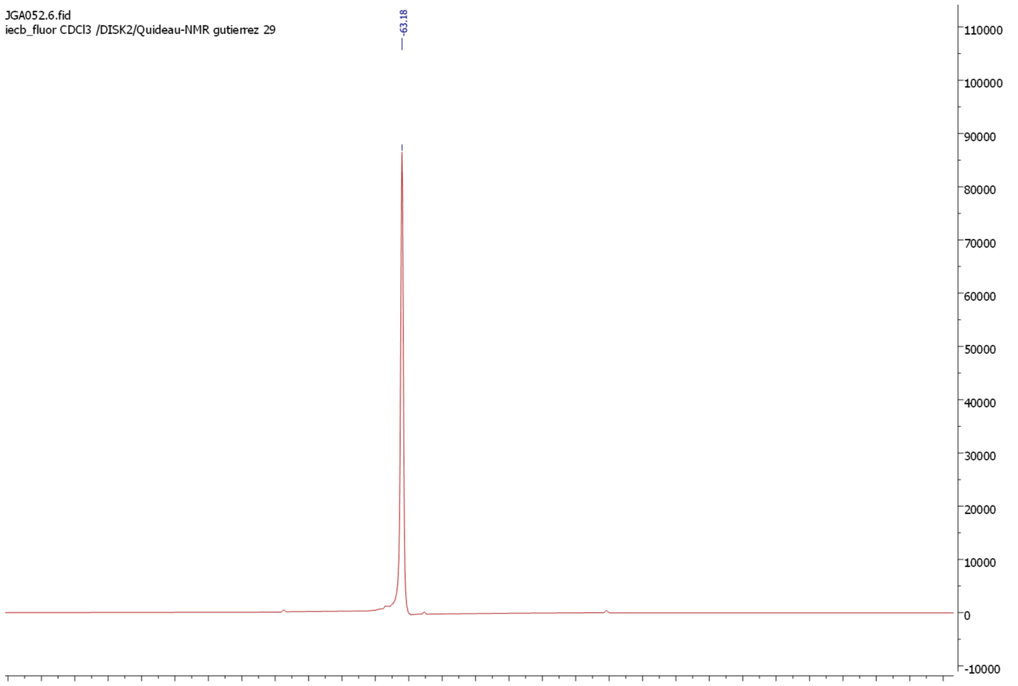

JGA052.6.fid
iecb_fluor CDCl3 /DISK2/Quideau-NMR gutierrez 29
-63.18
110000
100000
90000
80000
70000
60000
50000
40000
30000
20000
10000
0
-10000
